# Transcription Factor CitERF16 Is Involved in Citrus Fruit Sucrose Accumulation by Activating *CitSWEET11d*

**DOI:** 10.3389/fpls.2021.809619

**Published:** 2021-12-23

**Authors:** Xiaobo Hu, Shaojia Li, Xiahui Lin, Heting Fang, Yanna Shi, Donald Grierson, Kunsong Chen

**Affiliations:** ^1^College of Agriculture & Biotechnology, Zhejiang University, Hangzhou, China; ^2^Zhejiang Provincial Key Laboratory of Horticultural Plant Integrative Biology, Zhejiang University, Hangzhou, China; ^3^The State Agriculture Ministry Laboratory of Horticultural Plant Growth, Development and Quality Improvement, Zhejiang University, Hangzhou, China; ^4^Division of Plant and Crop Sciences, School of Biosciences, University of Nottingham, Nottingham, United Kingdom

**Keywords:** citrus fruit, sucrose, SWEET transporters, transcriptional regulation, ERF

## Abstract

Sugars are the primary products of photosynthesis and play an important role in plant growth and development. They contribute to sweetness and flavor of fleshy fruits and are pivotal to fruit quality, and their translocation and allocation are mainly dependent on sugar transporters. Genome-wide characterization of Satsuma mandarin identified eighteen *SWEET* family members that encode transporters which facilitate diffusion of sugar across cell membranes. Analysis of the expression profiles in tissues of mandarin fruit at different developmental stages showed that *CitSWEET11d* transcripts were significantly correlated with sucrose accumulation. Further studies indicated that overexpression of *CitSWEET11d* in citrus callus and tomato fruit showed a higher sucrose level compared to wild-type, suggesting that CitSWEET11d could enhance sucrose accumulation. In addition, we identified an ERF transcription factor CitERF16 by yeast one-hybrid screening assay which could directly bind to the DRE *cis*-element on the promoter of *CitSWEET11d*. Overexpression of *CitERF16* in citrus callus significantly induced *CitSWEET11d* expression and elevated sucrose content, suggesting that CitERF16 acts as a positive regulator to promote sucrose accumulation *via* trans-activation of *CitSWEET11d* expression.

## Introduction

Sugars are the primary products of photosynthesis and play an important role during plant growth and development as sources of carbon skeletons, osmolytes, signals, transport molecules, and transient energy storage (Chen et al., 2015b). The assimilate is usually synthesized in leaves (source tissues) and then transported to other non-photosynthesis organs (sink tissues), such as root, seed, flower, and fruit to support their growth and development (Baker et al., 2012; Chen, 2014; Ruan, 2014).

For fruit, sugar is a major contributor to the sweetness which is one of the most important fruit organoleptic qualities (Wei et al., 2014). Sugar partitioning is crucial in sugar accumulation since fruit usually accumulates high level of sugars, especially sucrose. Sucrose is the main carbon form transported from source to sink and supplies carbon source and energy for plant growth and development (Ruan, 2014; Julius et al., 2017). In addition, sucrose acts as an important signaling molecule and has been implicated in physiological processes and response to biotic and abiotic stresses (Ruan et al., 2010; Lastdrager et al., 2014; Wei et al., 2018). Sucrose translocation and accumulation usually rely on transporters which are the essential regulators for sucrose transport (Slewinski, 2011). To date, there exist two key families of sucrose transporters, SUTs (sucrose transporters) and SWEETs (Sugar Will Eventually be Exported Transporters). SoSUT1 from spinach was the first sucrose transporter identified in plants and the *SUT* family has been studied for nearly thirty years. Numerous reports have investigated and described the functional roles of SUTs. It has been found that SUTs are mainly located in the plasma membrane and are widely involved in phloem loading, unloading, carbohydrate distribution, storage, and reproductive development (Sun et al., 2019a). SWEETs have been identified as sugar transporters more recently, by heterologous co-expression with high-sensitivity fluorescence resonance energy transfer (FRET) sensors in human HEK293T cells (Chen et al., 2010; Eom et al., 2015). They function as uniporters and facilitate diffusion of sugars across cell membranes down a concentration gradient (Baker et al., 2012; Braun, 2012; Chen, 2014). In recent years, *SWEET* family members have been principally identified in model plants, such as *Arabidopsis thaliana*, rice (*Oryza sativa*), and tomato (*Solanum lycopersicum*; Chen et al., 2010; Yuan et al., 2014; Feng et al., 2015), and their roles in diverse physiological processes, such as phloem loading, nectar secretion, seed filling, and host-pathogen interactions, by mediating hexose and sucrose transport have been well characterized (Chen et al., 2012; Yuan and Wang, 2013; Lin et al., 2014; Yang et al., 2018). In *Arabidopsis*, overexpression of *AtSWEET4* caused larger plants, which accumulated more glucose and fructose, and exhibited enhanced freezing tolerance (Liu et al., 2016). In rice, *OsSWEET11* and *OsSWEET15* were highly expressed in caryopses and both genes were necessary for seed filling (Yang et al., 2018). In addition, SWEETs are also the targets of bacteria and fungi as they provide the necessary nutrition for their growth and reproduction (Chong et al., 2014). For fruit, the accumulation of sugar is a most important feature which determines the quality and yield. Although there have been numerous studies in which *SWEET* members were identified at the genome level (Chong et al., 2014; Li et al., 2017a; Guo et al., 2018), compared to their well-known functions in phloem loading and seed filling described above, the understanding about the roles of SWEET in fruit development and sugar accumulation remains limited. In tomato, SlSWEET1a located in the plasma membrane could increase fructose level and modify the composition of sugar in fleshy fruit (Shammai et al., 2018). In pear, overexpression of *PuSWEET15* contributed to sucrose accumulation in pear fruit (Li et al., 2020b). However, the *SWEET* family has not been explored in citrus and their potential roles in citrus fruit require functional characterization. The exploration in citrus will not only advance and improve our understanding of SWEET functions in sugar accumulation but also provide new target genes for future genetic breeding, which will be of great significance to fruit quality improvement.

Although there has been considerable progress related to SWEETs in various species, including extensive research in model plants (Chen et al., 2015a; Ma et al., 2017; Sun et al., 2019b), the underlying regulatory mechanisms controlling their expression are still poorly understood. Recently, there have been some clues suggesting that *SWEETs* could be regulated at the transcriptional level and post-translational level. SOC1, which encodes a MADS box transcription factor, might act as a mediator to activate the transcription of *SWEET10*, a sucrose transporter causing early flowering and response to long days (Andres et al., 2020). PuWRKY31 has been reported to activate the expression of *PuSWEET15*, a member of clade III, and elevated sucrose level in pear fruit (Li et al., 2020b). Transcription activator-like (TAL) effectors act as transcription factors regulating the transcript abundance of *SWEETs*, so that the sugar effluxed into the apoplast can be utilized by pathogens (Streubel et al., 2013; Bezrutczyk et al., 2018). In potato, the interaction between StSWEET11 and StSP6A blocked the sucrose leakage into the apoplast, leading to a shift from apoplasmic unloading to symplasmic unloading (Abelenda et al., 2019). Nevertheless, the specific mechanisms remain unclear and further exploration of the regulation of *SWEETs* is necessary to elucidate and understand the regulatory network governing production and activity of SWEETs.

In the current study, eighteen *SWEET* members were identified in Satsuma mandarin based on genome-wide analysis, their expression profiles during fruit development were analyzed and the transcript level of *CitSWEET11d* was found to be highly positively correlated with sucrose accumulation. Overexpression of *CitSWEET11d* in both citrus callus and tomato fruit caused higher levels of sucrose, suggesting that CitSWEET11d contributed to sucrose accumulation, which has provided new candidate for improving fruit quality during breeding. Furthermore, a transcription factor CitERF16 was identified by yeast one-hybrid screening. Electrophoretic mobility shift assay (EMSA) and dual-luciferase assays showed that it could directly bind to the promoter of *CitSWEET11d* and induce its expression. Overexpression of *CitERF16* in citrus callus significantly elevated the sucrose content, probably by regulating *CitSWEET11d* transcripts. In summary, our results enrich and broaden the regulatory network of manipulating *SWEETs* and provide new insights into the functional roles of SWEETs to contribute to fruit quality by mediating sucrose accumulation.

## Materials and Methods

### Plant Materials

Fruits, roots, stems, leaves, and flowers of Satsuma mandarin (*Citrus unshiu* Marcov. Variety Miyagawa wase) were obtained from Linhai, Zhejiang province, China. Uniform fruits were harvested at five time points, 60, 90, 120, 150, and 180 DAFB (day after full bloom) with three biological replicates. Fruit flesh and other tissue samples were frozen in liquid nitrogen, and stored at −80°C until further use. Similarly, the fruits of various citrus varieties with three biological replicates were harvested at the mature stage and the frozen flesh was stored at −80°C. “TK,” “JG,” “GT,” “STL,” and “WZ” represent the names of different varieties, “TianKou,” “JingGang 1,080,” “GuTian,” “ShouTaiLang,” and “WeiZhang,” respectively.

*Solanum lycopersicum* Alisa Craig (AC) was used in this study and grown in the greenhouse at 25°C (16 h light/8 h dark). The ripe fruits (seven days after breaker stage) were collected. Pericarp was cut into pieces, frozen in liquid nitrogen, and stored at −80°C.

Valencia orange embryogenic callus in tissue culture was used for transformation and grown on MT medium containing 2% sucrose supplemented with 5 mg l^−1^ vitamin C at 25°C under long-day conditions (16 h light/8 h dark). After infection mediated by agrobacterium, 50 mg l^−1^ Kanamycin was added into the medium for selection. They were subcultured at 30-day intervals.

### Identification and Sequence Analysis of the Citrus SWEET Family

The SWEET family genes were identified *via* BLASTP searches of the national center for biotechnology information (NCBI) and citrus genome database,^1^ using AtSWEETs protein sequences as queries, and 18 CitSWEETs with MtN3_saliva domain were identified. The gene IDs of the *SWEET* family in citrus are listed in Supplementary Table S2 and those from *Arabidopsis* and rice were obtained from Chen et al. (2010). The corresponding amino acid sequences of SWEET proteins in *Arabidopsis* and rice were acquired from The Arabidopsis Information Resource and Rice Genome Annotation Project and then utilized to construct the phylogenetic tree. The neighbor-joining method was exploited to align the proteins in Clustal X (version 1.8.1), and the phylogenetic tree was constructed using FigTree with default parameters (version 1.3.1). Transform branches were selected to generate the tree, and adjust other minor parameters as needed, such as fonts and line weight.

The exon-intron structure and intron phases were acquired by the online Gene Structure Display Server 2.0.^2^ The intron phases are defined as shown below: intron inserted between two codons is described as phase 0, intron inserted between first and second base of a codon is described as phase 1, and an intron inserted between second and third base is described as phase 2.

The conserved motifs among SWEETs were identified, based on their amino acid sequences, by the MEME tool. The optimized parameters of MEME have been described in previous studies (Guo et al., 2018).

### Sugar Content Measurements

The sugars of peeled citrus fruit were extracted and measured according to Li et al. (2017b) with some modification. Each sample was weighed and 0.1 g placed in 1.4 ml chromatographic methanol at 70°C. After 15 min, the sample was centrifuged for 10 min at 10000 g. The upper phase was collected and mixed with 1.5 ml Mili-Q water and 0.75 ml trichloromethane. After centrifugation at 2200 g for 10 min, 100 ul supernatant was collected again, dried in a vacuum, and then dissolved in 60 ul 20 mg ml^−1^ pyridine methoxyamine hydrochloride for 1.5 h at 37°C. Next, the sample was mixed with 40 ul Bis (trimethylsilyl) trifluoroacetamide (1% trimethylchlorosilane) for 0.5 h at 37°C. Each sample contained 20 ul ribitol (0.2 mg ml^−1^), which was used as an internal standard. 1 ul final sample was taken and injected into a GC-MS fitted with a fused-silica capillary column (30 m × 0.25 mm internal diameter, 0.25 um DB-5 MS stationary phase). The injector temperature was 250°C and the helium carrier gas had a flow rate of 10.0 ml min^−1^. The column temperature was held at 100°C for 1 min, increased to 185°C at a rate of 2.5°C min^−1^, then increased to 190°C at a rate of 0.35°C min^−1^, subsequently increased to 250°C at a rate of 8°C min^−1^ and held for 5 min, then finally increased to 280°C at a rate of 5°C min^−1^ and held for 3 min. The ion source temperature was 230°C, and interface temperature was 280°C.

### RNA Extraction and Real-Time PCR

According to the protocol described in our previous report (Li et al., 2017b), total RNA from citrus flesh and tissues was extracted. After removing genomic DNA with RNase-free DNase I (Ambion), 1.0 ug DNA-free RNA was used to synthesize the cDNA using GoScript^™^ Reverse Transcriptase (Promega), then the obtained cDNA was diluted with water and used as template. Real-time PCR was conducted by a LightCycler 480 instrument (Roche) with LightCycler 480 SYBR Green I Master (Roche). Citrus actin (XM_006464503) was used as the housekeeping gene. Primers for RT-qPCR analysis are listed in Supplementary Table S3.

### Yeast One-Hybrid Assay

The interaction between the transcription factor and the promoter was confirmed by yeast one-hybrid assay using the Matchmaker Gold Yeast One-hybrid Library Screening System (Clotech). The promoter sequence of *CitSWEET11d*, obtained from Citrus Genome Database, was inserted into the pAbAi vector, and a prey cDNA library (TaKaRa) was constructed using total RNA from citrus fruit. Promoter auto-activation was detected on SD medium lacking Ura in the presence of aureobasidin A (AbA) according to the system manual. Further, the coding sequence of screened transcription factors was cloned and inserted into the pGADT7 vector and then the protein-DNA interaction was verified. The empty vector was used as the negative control. The primers are listed in Supplementary Table S4.

### Dual-Luciferase Assay

Dual-luciferase assays were carried out according to our previous report (Zhang et al., 2018). The transcription factor coding sequence was amplified and inserted into the pGreen II 0029 62-SK vector (SK) and the promoter of *CitSWEET11d* was constructed into the pGreen II 0800-LUC vector (LUC). The primers used for amplification are listed in Supplementary Table S4. The recombinant plasmids were individually electroporated into *Agrobacterium tumefaciens* GV3101. After culturing for 2–3 days, the *A. tumefaciens* were collected and suspended in infiltration buffer, adjusting the concentration of the suspension to an OD_600_ of 0.75, and mixing the *A. tumefaciens* containing TFs and promoters in a ratio of 10:1. Then the mixture was infiltrated into tobacco (*Nicotiana benthamiana*) leaves using a needleless syringe. The tobacco plants were grown in a greenhouse with a light/dark cycle of 16: 8 h at 25°C. Three days later, the enzyme activities of firefly luciferase and Renilla luciferase were measured with the Dual-luciferase Reporter Assay System (Promega). Three biological replicates were conducted for each TF-promoter interaction study.

### Subcellular Localization Analysis

The coding sequence of *CitSWEET11d* without the stop codon was amplified and inserted into pCAMBIA1300-sGFP vector to form *Pro-35S: CitSWEET11d-GFP*. The 35S-CitSWEET11d-GFP plasmid was introduced into *A. tumefaciens* (GV3101) and then co-expressed transiently with the vacuolar membrane marker (AtTIP-mCherry) into the leaves of transgenic tobacco which has been stably transformed with a specific nucleus-localized red fluorescent protein construct. The method was the same as that in dual-luciferase assay. After two days, the tobacco leaves were collected and imaged using a Zeiss LSM710NLO confocal laser scanning microscope. The primer used is listed in Supplementary Table S4.

### CitERF16 Recombinant Protein and EMSA Analysis

Recombinant protein and EMSA analysis were carried out as described in our previous report (Ge et al., 2017). The coding sequence of *CitERF16* without the stop codon was inserted into the pET-32a vector (Clontech). The recombinant plasmids were introduced into the *Escherichia coli* strains Rosetta 2(DE3)pLysS (Novagen, Germany), and the CitERF16 protein with His tags was expressed. 1 mm isopropyl β-D-1-thiogalactopyranoside was added to the liquid culture containing transformed cells and incubated at 37°C for 3 h. The cultures were centrifuged at 5000 g for 15 min (4°C), the collected cells were suspended in buffer (20 mm Tris-HCl, pH 8, 0.5 mm NaCl, 10 mm β-mercaptoethanol, and 10% glycerol) and then ultrasonicated in an ice bath at 200 W with a 3 s/2 s on/off cycle. 30 min later, the mixture was centrifuged at 9000 g for 20 min at 4°C. According to the instructions of the GST-tag Protein Purification Kit (Beyotime), the purified protein was confirmed using SDS-PAGE.

EMSA assay was carried out to verify the binding of recombinant protein and core sequences in the *CitSWEET11d* promoter. The biotinylated single-strand oligonucleotides were converted to dsDNA probes by annealing complementary oligonucleotides. The EMSA experiments were performed according to the manufacturer’s instructions in Lightshift Chemiluminescent EMSA kit (Thermo). The probes used are described in Supplementary Table S4.

### Agrobacterium-Mediated Transformation

The full-length *CitERF16* or *CitSWEET11d* were amplified and inserted into PBTEX vector to form *Pro-35S: CitSWEET11d* and *Pro-35S: CitERF16*, separately. The recombinant plasmids were transformed into *A. tumefaciens* strain EHA105 and the transformation assays were performed using the *Agrobacterium*-mediated method as described previously with minor modification (Li et al., 2002). After co-culture for 3 days in darkness, the callus was transferred to MT mediums containing 2% sucrose; 50 mg/l kanamycin and 400 mg/l cefotaxime were used for the next screening, and the callus was subcultured every one month and grown at 26°C under darkness until resistant callus formed. After propagating, twenty-day-old callus was harvested and stored at −80°C for further analysis.

### Generation of Transgenic Tomato Plants

The coding sequence of *CitSWEET11d* without stop codon was inserted into the pCAMBIA-1301 vector driven by the 35S promoter, fused with GUS reporter, then the plasmid was introduced into *A. tumefaciens* strain EHA105. The stable transformation of tomato was carried out as described in Li et al. (2018). Overexpressed lines were identified by PCR detection. T2 generation fruits were used for sugar and gene expression analysis.

### Heterologous Expression of *CitSWEET11d* in Mutant Yeast Strains

The open reading frame of *CitSWEET11d* was inserted into the PDR196 vector, then recombinant vectors were transformed into yeast mutant strains EBY.VW4000 and SUSY7/*ura3* according to the method described in Cheng et al. (2015), and yeast transformants with empty vector were used as the control. The primers used are listed in Supplementary Table S4. For yeast hexose mutant cells, the liquid SD medium lacking Ura complemented with 2% (w/v) maltose as the sole carbon source was used to culture the transformants to OD_600_ 0.6, then the cell suspensions were diluted to 0.06 and 0.006, different dilutions (×1, ×10, ×100) were dropped on solid SD media containing either 2% maltose (control), fructose or glucose without Ura. For yeast sucrose uptake-deficient cells, glucose was the sole carbon source in SD medium. After serial dilution, dilutions (×1, ×10, ×100) were dropped on solid mediums with 2% glucose (control) or 2% sucrose. Yeast cells were incubated at 30°C for 3–5 d. Three biological replicates were performed for individual experiment.

### Gus Staining

Histochemical staining was performed to confirm the co-expression of the GUS reporter and *CitSWEET11d* with the GUS stain kit (RealTimes). X-gluc solution and GUS staining buffer were mixed according to instructions. The leaves were cut into small pieces and submerged in the mixed solution for 10 min or even more time until the leaves became blue. After staining, 75% ethanol was used to eliminate the chlorophyll present in the stained leaves.

### Statistical Analysis

Standard errors and figures were made using Origin Pro 8.0 (Microcal Software, Inc., Northampton, MA, United States). LSD values at 5% level were calculated using Microsoft Excel. Significance was analyzed by Student’s t test (^*^p < 0.05, ^**^p < 0.01 and ^***^p < 0.001). Scatterplots were performed with GraphPad Prism 7.0 (GraphPad Software Inc., San Diego, CA, United States) and correlation coefficients were analyzed using Pearson method.

## Results

### Identification of *SWEET* Genes in Citrus

**Figure 1:**
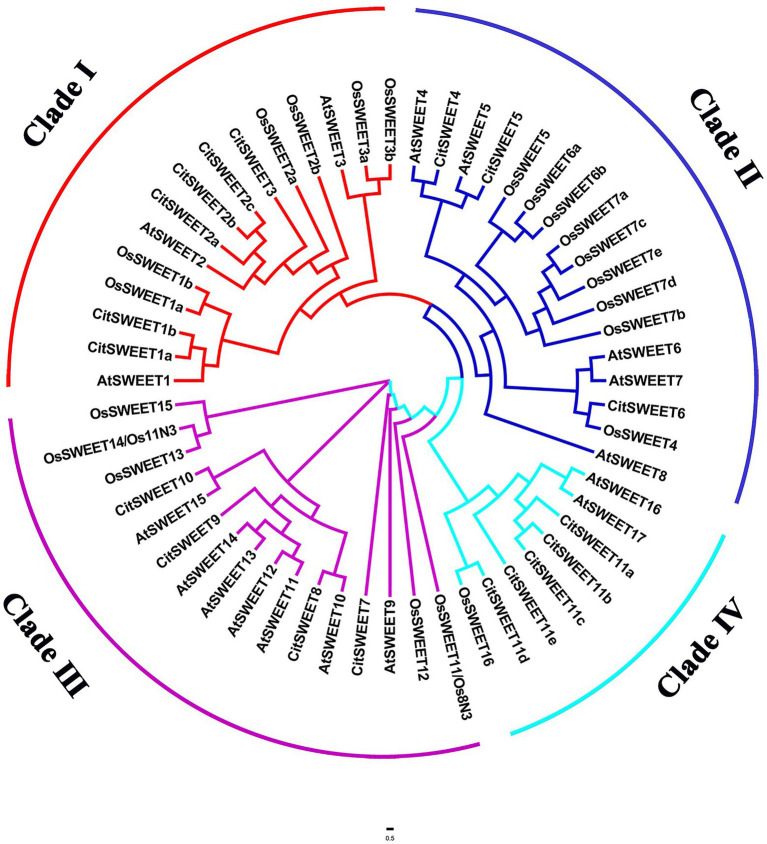
Phylogenetic analysis of SWEET family members from citrus, *Arabidopsis* and rice. The amino acid sequences were obtained from the National Center for Biotechnology Information (NCBI), Citrus Genome Database, The Arabidopsis Information Resource and Rice Genome Annotation Project databases. Different colors represent different clades. At: *Arabidopsis thaliana*, Os: *Oryza Sativa*, Cit: *Citrus Clementina*.

Eighteen *SWEET* genes with PFAM motif PF03083 were identified in Satsuma mandarin and named after their homologous SWEETs in *A*. *thaliana*. A phylogenetic tree was constructed by FigTree using protein sequences of 18 CitSWEETs, 17 AtSWEETs, and 21 OsSWEETs. *CitSWEETs* were clustered into four clades, with six members in clade I (*CitSWEET1a, b*; *CitSWEET2a, b, c*; *CitSWEET3*); three members (*CitSWEET4, 5, 6*) in clade II; four in clade III (*CitSWEET7, 8, 9, 10*); and five *SWEETs* in clade IV (*CitSWEET11a, b, c, d, e*; Figure 1; Supplementary Table S1).

### Sucrose Content and Expression Patterns of the *SWEET* Genes During Fruit Development in “Gongchuan” (*Citrus Unshiu* Marcov. Miyagawa Wase)

**Figure 2:**
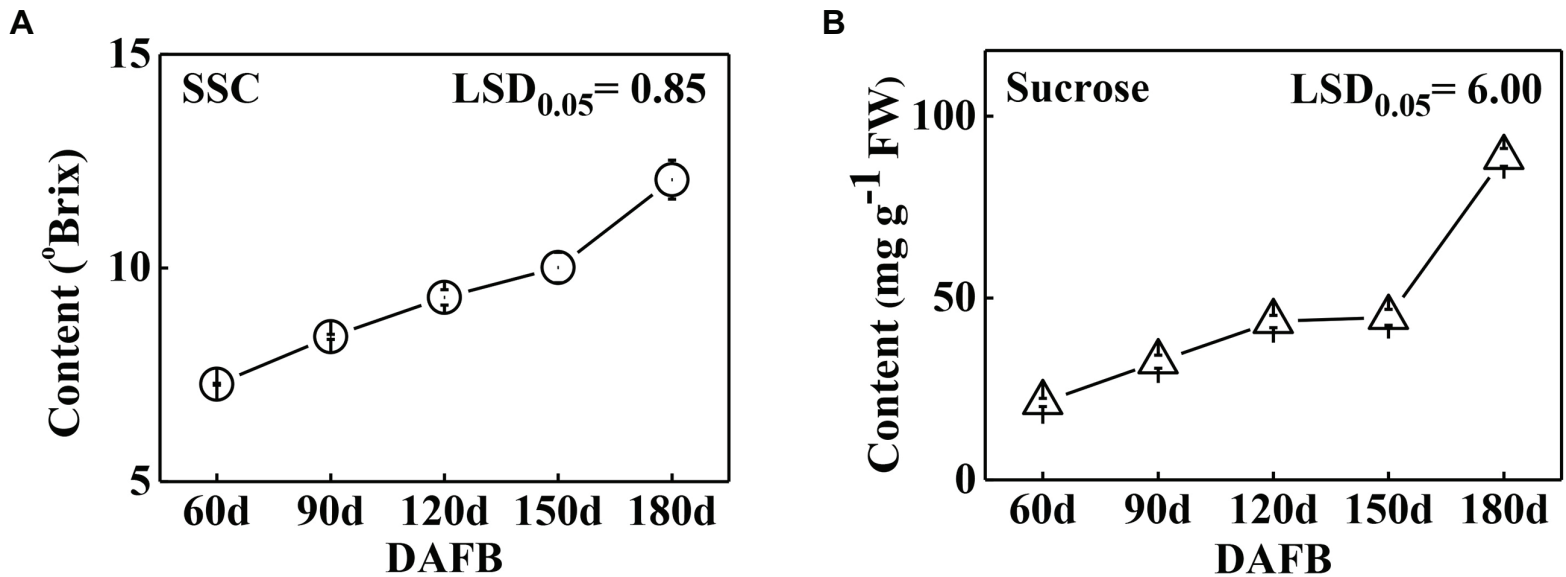
The soluble solids **(A)** and sucrose **(B)** contents during development in ‘Gongchuan’ citrus fruit. The fruits were harvested at different time points and peels were removed, then sucrose level was determined in fruit flesh. FW, fresh weight. Error bars represent ±SE from three biological replicates (*n* = 3). LSD values were calculated at *p* = 0.05.

**Figure 3:**
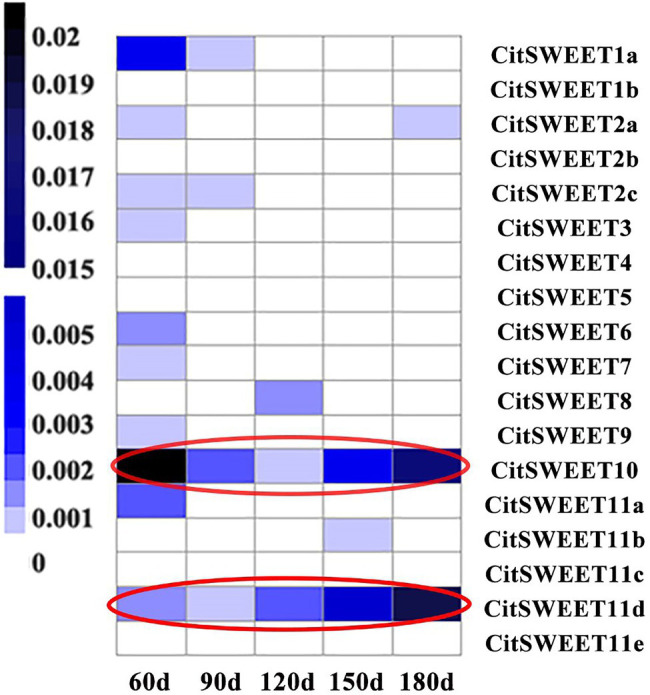
The expression analysis of *SWEET* family during citrus fruit development. The corresponding gene names are listed in the right-hand column. The letters under each sample column denote different development stages (DAFB). There were three biological replicates for each point and the average number was used to generate the heatmap. Transcript abundance is indicated by color, with the scale shown on the left.

The soluble solids were determined during Satsuma mandarin fruit development. The results showed that the soluble solids content gradually increased during development (Figure 2A), which increased relatively slowly at the earlier and middle stages of development (60 DAFB to 150 DAFB), while turned to rapid increase at the later stage of growth and maturation (150 DAFB to 180 DAFB). As sucrose is the most dominant sugar in citrus fruit, the sucrose content was further detected (Figure 2B). The results indicated that sucrose content showed a continuous increase during development, and the accumulation mainly occurred at the later stage (150 DAFB to 180 DAFB), which is consistent with the change of soluble solids content (Figure 2). To investigate the role of *SWEETs* in citrus fruit development, their expression patterns were analyzed. No *CitSWEET2b, CitSWEET5, CitSWEET11c*, and *CitSWEET11e* transcripts were found in fruit. The transcript abundance of *CitSWEET2a* remained at a similar level throughout development, whereas the transcripts of *CitSWEET1a, CitSWEET3, CitSWEET9*, and *CitSWEET11a* gradually decreased during fruit development and transcripts of *CitSWEET1b* could only be detected in the earliest stage (60d DAFB). Both *CitSWEET10* and *CitSWEET11d*, however, were highly expressed during fruit maturation. Correlation coefficients obtained by the Pearson method showed that the association of *CitSWEET10* with sucrose content was not significant (Supplementary Figure S4). In contrast, the transcript abundance of *CitSWEET11d* was consistent with the accumulation of sucrose, and the relative index was up to 0.93 (Figure 3; Supplementary Figure S4). This suggested that the function of *CitSWEET11d* might be closely related to sucrose accumulation.

### The Functional Characterization of *CitSWEET11d* in Sucrose Accumulation

The subcellular localization assay showed that CitSWEET11d was located at the tonoplast together with the tonoplast marker (Supplementary Figure S5). Heterologous expression of *CitSWEET11d* in yeast mutant cells was conducted to explore its potential transport properties. As shown in Supplementary Figure S6, when *CitSWEET11d* was expressed in sucrose transport-deficient yeast strain, SUSY7/*ura3*, the *CitSWEET11d*-SUSY7/*ura3* transformants were unable to restore growth on sucrose medium while the growth on glucose medium was normal. Furthermore, the hexose transport activities of CitSWEET11d were detected by expressing in yeast hexose mutant strain, EBY.VW4000 and similar results were observed that the growth on maltose medium was normal, but the cells were unable to grow on fructose or glucose medium.

**Figure 4:**
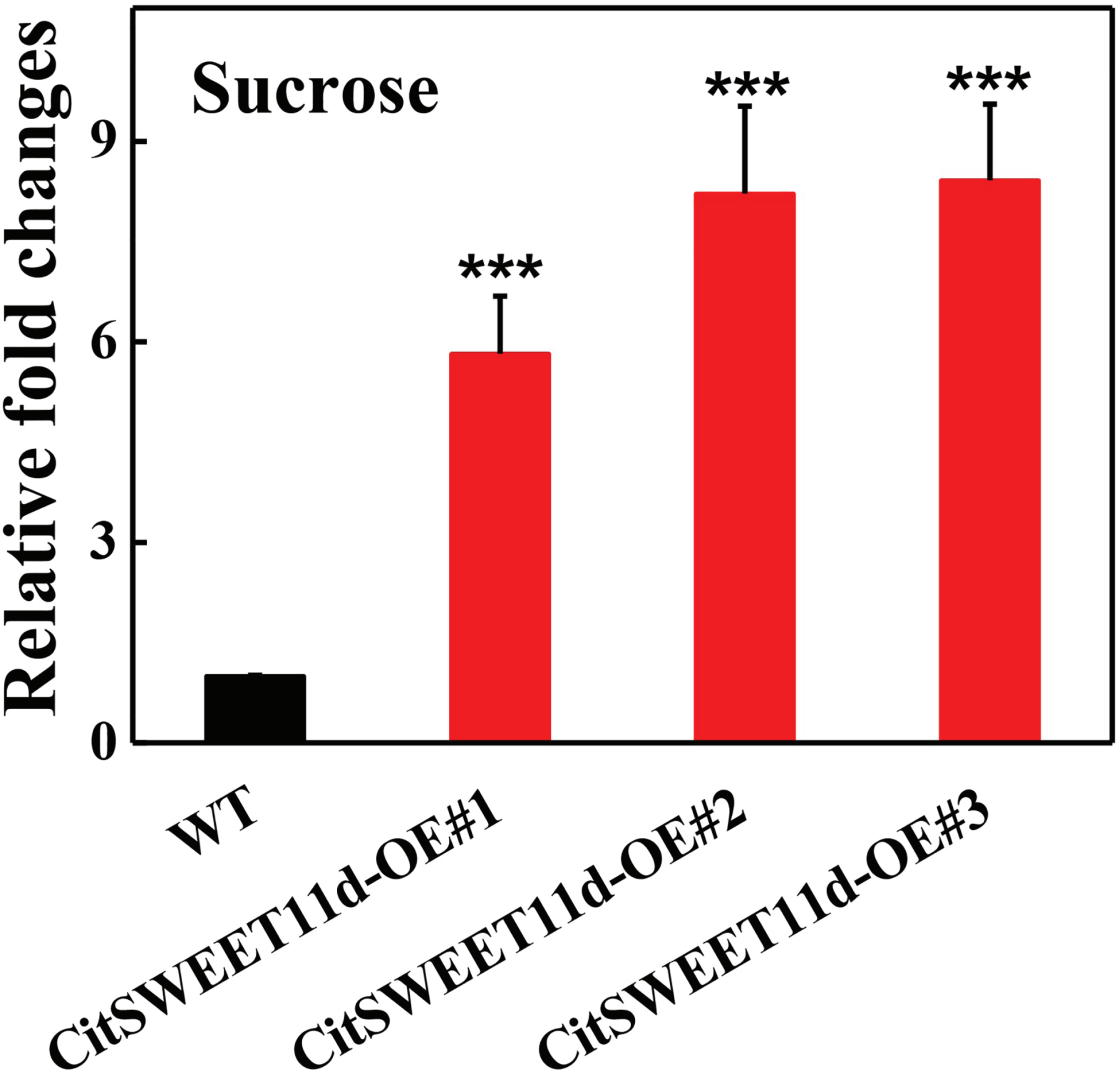
Relative fold changes of sucrose content in wild-type (WT) and callus overexpressing *CitSWEET11d*. Twenty-day-old callus was collected and used to determine sucrose content. The sucrose content in WT callus was set as 1, then those in overexpressing *CitSWEET11d* lines were converted into multiples. Error bars represent ±SE (*n* = 3). Statistical significance was determined by Student’s two-tailed t test (^***^p < 0.001).

To further validate the function of CitSWEET11d, we studied the effects on sucrose content by overexpressing it in citrus callus grown in tissue culture. Three independent transgenic lines, CitSWEET11d-OE#1, CitSWEET11d-OE#2 and CitSWEET11d-OE#3 were obtained and the sucrose contents were 5.8-fold, 8.2-fold and 8.4-fold higher, respectively, than that in wild-type callus grown in medium containing 2% sucrose, which confirmed that the transgenic lines accumulated higher level of sucrose than the wild-type callus (Figure 4). As citrus is a perennial plant, and genetic transformation is difficult and time-consuming, we used the model plant tomato as an alternative way of testing CitSWEET11d function. We overexpressed *CitSWEET11d* in tomato (Alisa Craig) and obtained two transgenic lines, CitSWEET11d-OE#19 and CitSWEET11d-OE#27. Transcript analysis and GUS staining assay demonstrated that *CitSWEET11d* has been successfully ectopically expressed in tomato (Supplementary Figure S8A). Furthermore, examination of the sugars in the ripe tomato fruit showed that sucrose contents in the two transgenic lines were 1.74 mg/g and 1.92 mg/g, which were 1.26-fold and 1.39-fold higher than that in wild-type fruit (1.37 mg/g), respectively, while no significant differences were observed for glucose and fructose (Supplementary Figure S8).

### Identification of CitERF16 and Its Interaction With the *CitSWEET11d* Promoter

**Figure 5:**
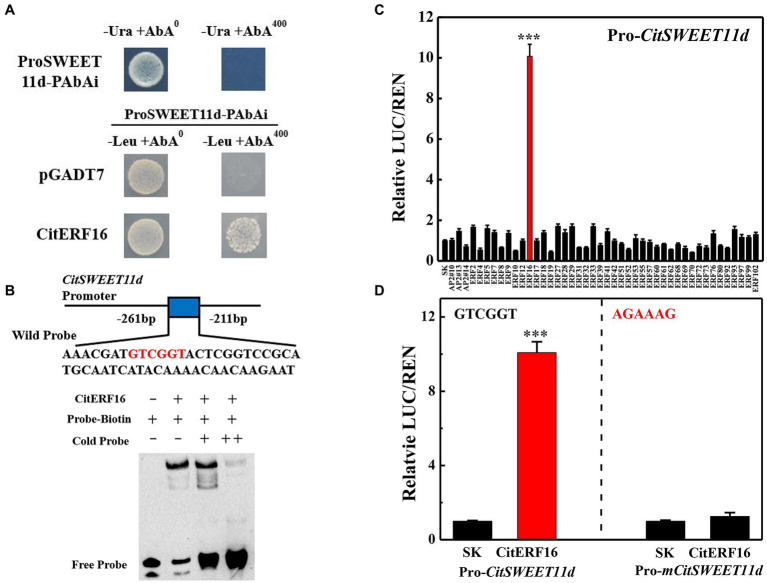
*In vivo* and *in vitro* interaction between CitERF16 and the target promoter of *CitSWEET11d*. **(A)** Yeast one-hybrid analysis of the interaction of CitERF16 with the *CitSWEET11d* promoter. An auto-activation test was performed on SD/-Ura medium in the presence of 400 ng/ml aureobasidin A (AbA). The physical interaction was determined on SD/−Leu medium in the presence of 400 ng/ml aureobasidin A (AbA). The empty pGADT7 vector was used as negative control. **(B)** EMSA of CitERF16 binding to the *CitSWEET11d* promoter. The binding core sequence in the promoter of *CitSWEET11d* is indicated in red. The 3'-biotin-labeled dsDNA probes were incubated with the purified CitERF16 protein. The symbols - and + represent absence or presence, respectively. **(C)** Regulatory effects of CitAP2/ERFs on the promoter of *CitSWEET11d* by dual-luciferase assay. The ratio of firefly luciferase and Renilla luciferase (LUC/REN), with the empty vector (SK) plus promoter was set as 1. **(D)** Regulatory effects of CitERF16 on the mutant promoter of *CitSWEET11d* (Pro-*mCitSWEET11d*) by dual-luciferase assay. The GTCGGT core sequence in the *CitSWEET11d* promoter was mutated to AGAAAG. Error bars indicate the SEs from three replicates. Statistical significance was determined by Student’s two-tailed t test (^***^p < 0.001).

In order to explore the transcriptional regulation of *CitSWEET11d*, a yeast library screening assay was conducted, using *CitSWEET11d* as a bait. 101 colonies were obtained, among which one ERF (CitERF16) transcription factor was identified. Yeast one-hybrid experiments were conducted to confirm the interaction between CitERF16 and the *CitSWEET11d* promoter under 400 ng/ml AbA (Figure 5A). Moreover, EMSA assay showed that a specific DNA-CitERF16 protein complex was detected when GTCGTT-containing oligonucleotides were used as labeled probes. When the concentration of cold probes was markedly increased, a reduction in binding of the biotinylated probes was observed (Figure 5B). Dual-luciferase experiments were performed to find out the potential regulation of the *CitSWEET11d* promoter by CitERF16 and 43 other ERFs members identified in citrus. The results showed that CitERF16 exhibited the highest effect, up to 10-fold activation, compared with the control, whereas no other ERFs tested gave an activation above 2-fold (Figure 5C). In addition, when we mutated the DRE motif (5'-GTCGTT-3') in the promoter of *CitSWEET11d*, the trans-activating effect of CitERF16 was abolished (Figure 5D). These results demonstrated the specific interaction between CitERF16 and the *CitSWEET11d via* the *cis-*element, DRE motif (5'-GTCGTT-3') within the promoter of *CitSWEET11d*.

### The Expression Pattern of *CitERF16* and Correlation Analysis

**Figure 6:**
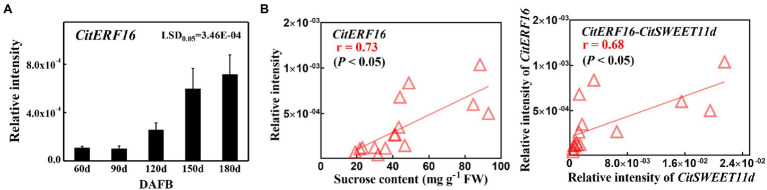
The *CitERF16* transcripts during citrus fruit development and correlation analysis with sucrose content and *CitSWEET11d* transcripts. **(A)** Expression level of *CitERF16* was determined at different fruit development stages. **(B)** Linear regression analysis between *CitERF16* expression and sucrose content (left) or *CitSWEET11d* expression (right). FW, fresh weight. Error bars represent ±SE (*n* = 3). LSD values were calculated at *p* = 0.05. Significant differences were determined by SPSS Statistics 20.0.

**Figure 7:**
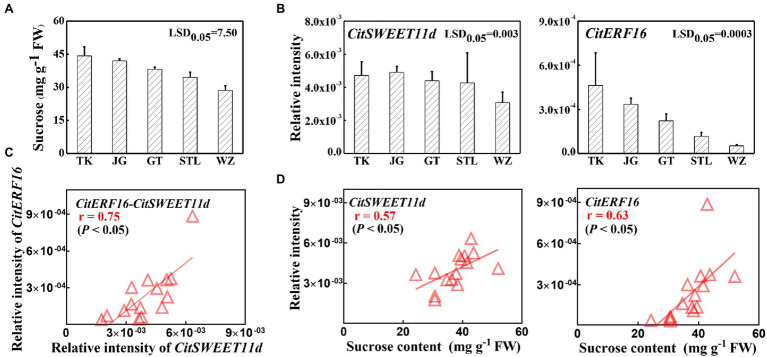
The sucrose content and correlation with *SWEET* and *ERF* genes transcripts in various citrus varieties. **(A)** Sucrose contents in different citrus varieties. **(B)** The expression levels of *CitSWEET11d* and *CitERF16* in different citrus varieties. **(C)** Linear regression analysis between *CitERF16* expression and *CitSWEET11d* expression. **(D)** Linear regression analysis between *CitSWEET11d* expression as well as *CitERF16* expression and sucrose contents. “TK,” “JG,” “GT,” “STL,” and “WZ” represent the names of different varieties, “TianKou,” “JingGang 1,080,” “GuTian,” “ShouTaiLang,” and “WeiZhang,” respectively. Error bars mean SEs from three biological replicates. LSD values were calculated at *p* = 0.05. Significant differences were determined by SPSS Statistics 20.0. FW, fresh weight.

The above results confirmed that CitERF16 could positively regulate *CitSWEET11d* by directly binding to the promoter. We analyzed the expression profile of *CitERF16* during fruit development, which showed a similar pattern with *CitSWEET11d*, and was consistent with the changes in sucrose content (Figure 6A). Linear regression analysis between the transcript level of *CitERF16* and the sucrose content as well as the transcript level of *CitSWEET11d* indicated that the correlations were significant (*p* < 0.05; Figure 6B). A highly consistent correlation was also observed in five different citrus varieties, among which the transcript abundance of both *CitSWEET11d* and *CitERF16* corresponded with sucrose content (Figures 7A,B). The further analysis showed that there existed a dramatically positive correlation between the expression level of *CitSWEET11d* and *CitERF16* (*p* < 0.05; Figure 7C). Moreover, the correlations between sucrose contents and the transcripts of the two genes were also significant (Figure 7D). These results suggest that CitERF16 may promote sucrose accumulation by regulating the transcripts accumulation of *CitSWEET11d*.

### Overexpression of *CitERF16* Promotes Sucrose Accumulation in Citrus Callus

**Figure 8:**
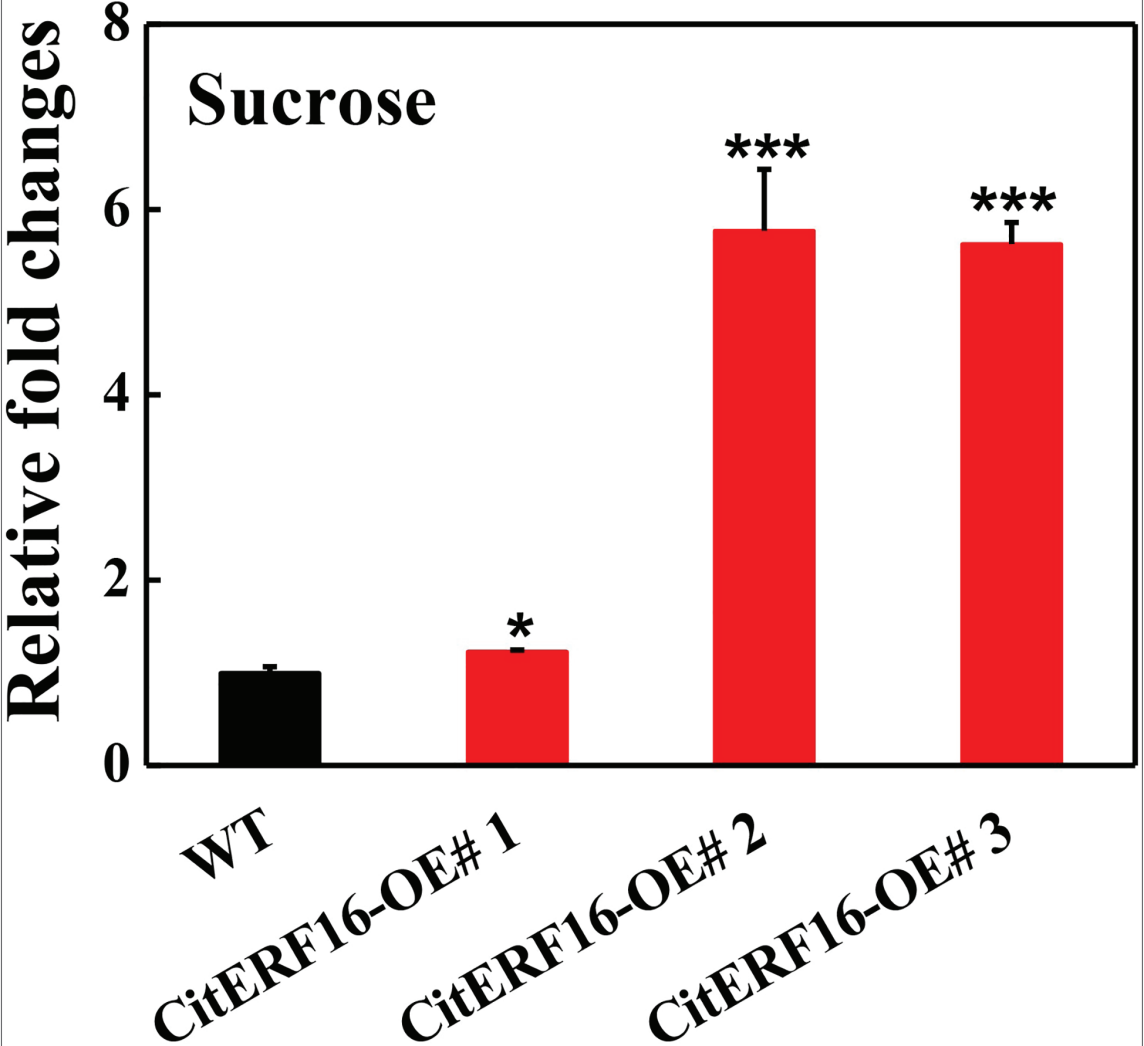
Relative fold changes of sucrose content in wild-type (WT) and callus overexpressing *CitERF16*. Twenty-day-old callus was collected and used to determine sucrose content. The sucrose content in WT callus was set as 1, then those in overexpressing *CitERF16* lines were converted into multiples. Error bars represent ±SE (*n* = 3). Statistical significance was determined by Student’s two-tailed t test (^*^p < 0.05; ^***^p < 0.001).

To further investigate its function, we overexpressed *CitERF16* in citrus callus and obtained three independent transgenic lines. The sucrose content in the callus grown in medium containing 2% sucrose was measured and the result showed that the sucrose level in wild-type callus was 3.13 mg/g, whereas significantly larger amounts of sucrose accumulated in overexpression lines (3.84 mg/g, 18.1 mg/g and 17.6 mg/g), which were 1.2-fold, 5.8-fold, 5.6-fold higher, respectively, than that in wild-type callus (Figure 8). In addition, the expression of *CitSWEET11d* was significantly induced in callus overexpressing *CitERF16*, compared with that in wild-type (Supplementary Figure S9B).

## Discussion

### Characterization of the *SWEET* Family in Citrus

*SWEET* genes are extensively found in various organisms, including plants, animals, fungi, and bacteria, and the *SWEET* family has been identified and characterized at the genome level in different species (Chen et al., 2010; Chong et al., 2014; Yuan et al., 2014; Feng et al., 2015). However, no systematic investigation of the *SWEET* family in Satsuma mandarin has been reported to date. Here, we isolated 18 *SWEET* members with an analysis of their phylogeny, gene structures, conserved motifs, and spatiotemporal expression patterns (Figure 1; Supplementary Figures S1–S3). The exon/intron distribution and the protein motifs were relatively conserved when compared to their paralogs. Generally, the acquisition or loss of introns leads to the generation of complex structures, which is considered as key evolutionary mechanism for most gene families (Xu et al., 2012). As shown in Supplementary Figure S1, most *CitSWEETs* have five introns, as in soybean and pineapple (Patil et al., 2015; Guo et al., 2018), while the average intron number is less than five in citrus. Combined with phylogenetic results, intron loss seems to be common in the clade I subfamily, in which approximately half of members have lost introns, particularly *CitSWEET2c*. In contrast, only one member has lost introns in other subfamilies, *CitSWEET6*, *CitSWEET7*, *CitSWEET11c* (Supplementary Figure S1). From this point of view, the gene structures in clade I subfamily are more diverse and complex, which may contribute to broader diversity and biological functions (Rogozin et al., 2005; Xu et al., 2012). In addition, loss of introns is usually accompanied by the loss of conserved motifs. Overall, the members in clade II subfamily have the most complete conserved motif structure, while motifs are missing in other subfamilies (Supplementary Figure S2). Model plants, such as *Arabidopsis* and rice, have only 2 and 1 *SWEETs* in clade IV, respectively, whereas the number in citrus is five, including *CitSWEET11d* (Supplementary Table S1). The more members of clade IV in citrus suggest that they may have developed more physiological roles in plant growth and development, which needs to be further explored.

As mentioned above, the *SWEET* genes are important for plant growth and development. The spatiotemporal expression patterns of citrus *SWEET* family showed that transcripts of more than half of the *SWEET* members could be detected in various organs (root, stem, leaf, flower), which is consistent with the conclusion that the *SWEET* family plays crucial roles in diverse physiological and biochemical processes (Chen, 2014; Chen et al., 2015b). Notably, all *CitSWEET* genes can be expressed at different levels in flowers, indicating that SWEETs are highly implicated in plant reproductive development in citrus, and similar result has been reported before (Jeena et al., 2019). For example, *CitSWEET7* had the highest expression level in flower and the transcripts in other tissues (root, stem, and leaf) were extremely low or negligible (Supplementary Figure S3), while its orthologous gene, *AtSWEET9*, was reported to encode a sucrose efflux transporter, essential for nectar production and secretion (Lin et al., 2014), so we speculate that the CitSWEET7 may also act as efflux transporter in nectar secretion. AtSWEET1, which has been described as a glucose uniporter, has two homologous genes in citrus, *CitSWEET1a* and *CitSWEET1b* (Chen et al., 2010). Their expression patterns in various organs were strikingly consistent and it is possible that there exists functional redundancy between the two paralogs (Supplementary Figure S3).

### CitSWEET11d Promotes Sucrose Accumulation in Citrus

SWEETs have been reported to be versatile transporters, capable of translocating glucose, fructose, and sucrose. For instance, the Arabidopsis AtSWEET1 is a glucose uniporter located in the plasma membrane in flowers and AtSWEET2 is a vacuolar sugar transporter highly expressed in root and accounts for glucose transport (Chen et al., 2010, 2015a). AtSWEET11 and AtSWEET12 are thought to be essential during the process in which sucrose flows out from phloem parenchyma cells, and are responsible for proper vascular development and cell wall composition (Le Hir et al., 2015). In addition, some SWEETs may act as multifunctional sugar transporters. For example, AtSWEET4 could mediate the transport of both glucose and fructose and AtSWEET16 has been identified as a tonoplast sugar transporter that can transport glucose, fructose, and sucrose (Klemens et al., 2013; Liu et al., 2016). In the present study, we identified a CitSWEET11d which was positively correlated with sucrose accumulation. A similar result was found in pear fruit, where PuSWEET15 was thought to be an essential sucrose transporter for sucrose accumulation (Li et al., 2020b). We carried out yeast sugar transport mutant complementation assays in order to study the potential transport roles of CitSWEET11d and the results indicated that *CitSWEET11d* could not complement the growth of yeast sucrose- or hexose-uptake mutants (Supplementary Figure S6). The cytoplasmic membrane localization of PuSWEET15, however, was the prerequisite for the success of yeast sucrose mutant assays (Li et al., 2020b). Based on this evidence, the possibility that CitSWEET11d acts as a functional transporter could not be definitively excluded (Bezrutczyk et al., 2018). Further work with a citrus transformation system showed that overexpression of *CitSWEET11d* in citrus callus could significantly elevate sucrose content (Figure 4). Moreover, overexpression of *CitSWEET11d* in tomato fruit resulted in a higher level of sucrose compared with the wild-type fruit, but fructose and glucose contents were not significantly altered (Supplementary Figure S8). Thus, it is reasonable to propose that CitSWEET11d might play a major role in sucrose accumulation in citrus and tomato.

It is known that plant SWEETs are not only localized at the plasma membrane, but also in Golgi and vacuole membranes and different members can transport sucrose, glucose, or fructose (Chen, 2014; Feng et al., 2015). Here, we found that CitSWEET11d was located at the tonoplast and shared the highest homology with AtSWEET17, followed by AtSWEET16 in *Arabidopsis*, which were identified as vacuolar transporters (Supplementary Figure S5; Chardon et al., 2013; Klemens et al., 2013). However, it is interesting that their biological roles greatly differ in some aspects. AtSWEET17 was principally expressed in root and stem and functioned as a fructose transporter to efflux fructose from the vacuole of the root and leaf to maintain the fructose homeostasis in *Arabidopsis* (Chardon et al., 2013; Guo et al., 2014). In contrast, CitSWEET11d contributed to sucrose accumulation rather than fructose accumulation in citrus. It is worth noting that in model plants and other species, the members of clade III, such as AtSWEET11, 12, OsSWEET11, and GmSWEET15, are mainly responsible for sucrose transport (Chen et al., 2015c; Ma et al., 2017; Wang et al., 2019), while the Clade IV members mainly account for hexose transport (Chen et al., 2015b). In citrus fruit, although *CitSWEET11d* belongs to Clade IV, it is involved in sucrose accumulation.

### CitERF16 Acts as a Novel Regulator for Sucrose Accumulation *via* Activating *CitSWEET11d*

More and more functional roles of the SWEET have recently been uncovered in plants (Guo et al., 2014; Yang et al., 2018; Sun et al., 2019b; Li et al., 2020b). Nonetheless, our understanding of the regulatory mechanism governing expression of *SWEETs* is still limited. Recent studies have shown that transcription factors are involved in regulating *SWEETs*. In rice, OsDOF11 can modulate sugar transport by regulating the expression of both *SUT* and *SWEET* genes (Wu et al., 2018); GhMYB212 regulates sucrose transport from the outer seed coat into the fibers by controlling the expression of *GhSWEET12* (Sun et al., 2019b). More recently, PuWRKY31 has been reported to bind to the *PuSWEET15* promoter and induce its transcription, thus promoting sucrose accumulation in pear fruit (Li et al., 2020b). In the present study, we identified an ethylene response factor CitERF16 by Y1H library screening, which could recognize and bind to the *cis*-element within the promoter of the *CitSWEET11d* and trans-activate the promoter activity (Figures 5A,C). The results were confirmed by EMSA analysis and dual-luciferase DNA mutation assays (Figures 5B,D). In addition, the expression profile of *CitERF16* was consistent with the expression of *CitSWEET11d* during fruit development and different citrus cultivars (Figures 6,7). These results suggest that CitERF16 is a novel type of activator of *CitSWEET11d*, which might broaden the range of transcription factors involved in modulating *SWEETs*.

AP2/ERFs are implicated in diverse biological processes, such as plant growth, development, and responses, to environmental stresses (Licausi et al., 2013; Jung et al., 2017; Xie et al., 2019). In recent years, ERF transcription factors have been extensively studied in various fruits and shown to have a range of functions that affect fruit quality, such as color (Li et al., 2019; An et al., 2020), volatile (Liu et al., 2017; Zhang et al., 2018), fruit acidity (Li et al., 2020a), and texture (Zhang et al., 2020). Our results showed that the expression of *CitERF16* was positively correlated with sucrose content during fruit development and in different citrus cultivars (Figures 6, 7) and sucrose content increased when overexpressing *CitERF16* in citrus callus (Figure 8), thus we propose that CitERF16 acts as a novel regulator for sucrose accumulation. Besides, previous studies have indicated that ERF transcription factors are associated with sucrose (Gilmour et al., 2000; Vogel et al., 2012; Huang et al., 2016). *CitERF16* was most closely related to the *DREB* genes, such as *CBF1-4* in *Arabidopsis*, which have been shown to participate in response to chilling and abscisic acid (Chen et al., 2019; Gratkowska-Zmuda et al., 2020). Overexpression of *CBFs* in different species, such as *Arabidopsis* and tomato, resulted in an increase in chilling or freezing tolerance (Hsieh et al., 2002; Gilmour et al., 2004). In addition, plants overexpressing *CBFs* have also increased sucrose levels (Knight and Knight, 2012). It is well-known that sucrose is an important contributor to cold tolerance, and sucrose content greatly increases in response to chilling (Wingler et al., 2015). Here, we have provided additional evidence that an ERF transcription factor regulated sucrose content in fruit.

## Conclusion

In summary, the citrus fruit *CitSWEETs* were characterized in the present study, and CitSWEET11d was found to be responsible for sucrose accumulation. In addition, we identified a novel transcription factor CitERF16, which could promote sucrose accumulation *via* transactivating *CitSWEET11d* promoter. These findings provide an important clue for underlying regulatory mechanism of *SWEETs* and new insights into how to manipulate sucrose accumulation in citrus and other fruits.

## Data Availability Statement

The datasets presented in this study can be found in online repositories. The names of the repository/repositories and accession number(s) can be found in the article/Supplementary Material.

## Author Contributions

KC and SL conceived the research plans and supervised the experiments. XH, XL, and HF performed the experiments and analysis. XH and SL wrote the article. YS and DG were involved in revising the manuscript. All authors read and approved the final article.

## Funding

This work was supported by National Key Research and Development Program (2016YFD0400101), the Fundamental Research Funds for the Central Universities (2021QNA6015), the National Natural Science Foundation of China (31801591), and the 111 project (B17039).

## Conflict of Interest

The authors declare that the research was conducted in the absence of any commercial or financial relationships that could be construed as a potential conflict of interest.

## Publisher’s Note

All claims expressed in this article are solely those of the authors and do not necessarily represent those of their affiliated organizations, or those of the publisher, the editors and the reviewers. Any product that may be evaluated in this article, or claim that may be made by its manufacturer, is not guaranteed or endorsed by the publisher.

## Footnotes

^1^ https://www.citrusgenomedb.org/

^2^ http://gsds.cbi.pku.edu.cn/

## References

[ref1] Abelenda J. A. Bergonzi S. Oortwijn M. Sonnewald S. Du M. Visser R. G. F.. (2019). Source-sink regulation is mediated by interaction of an FT homolog with a SWEET protein in potato. Curr. Biol. 29, 1178.e6–1186.e6. doi: 10.1016/j.cub.2019.02.018, PMID: 30905604

[ref2] An J. P. Zhang X. W. Bi S. Q. You C. X. Wang X. F. Hao Y. J. (2020). The ERF transcription factor MdERF38 promotes drought stress-induced anthocyanin biosynthesis in apple. Plant J. 101, 573–589. doi: 10.1111/tpj.14555, PMID: 31571281

[ref3] Andres F. Kinoshita A. Kalluri N. Fernandez V. Falavigna V. S. Cruz T. M. D.. (2020). The sugar transporter SWEET10 acts downstream of *FLOWERING LOCUS T* during floral transition of *Arabidopsis thaliana*. BMC Plant Biol. 20:53. doi: 10.1186/s12870-020-2266-0, PMID: 32013867 PMC6998834

[ref4] Baker R. F. Leach K. A. Braun D. M. (2012). SWEET as sugar: new sucrose effluxers in plants. Mol. Plant 5, 766–768. doi: 10.1093/mp/sss054 22815540

[ref5] Bezrutczyk M. Yang J. Eom J. S. Prior M. Sosso D. Hartwig T.. (2018). Sugar flux and signaling in plant-microbe interactions. Plant J. 93, 675–685. doi: 10.1111/tpj.13775, PMID: 29160592

[ref6] Braun D. M. (2012). SWEET! The pathway is complete. Science 335, 173–174. doi: 10.1126/science.1216828, PMID: 22246760

[ref7] Chardon F. Bedu M. Calenge F. Klemens P. A. Spinner L. Clement G.. (2013). Leaf fructose content is controlled by the vacuolar transporter SWEET17 in *Arabidopsis*. Curr. Biol. 23, 697–702. doi: 10.1016/j.cub.2013.03.021, PMID: 23583552

[ref8] Chen L. Q. (2014). SWEET sugar transporters for phloem transport and pathogen nutrition. New Phytol. 201, 1150–1155. doi: 10.1111/nph.12445, PMID: 24649486

[ref9] Chen T. Chen J. H. Zhang W. Yang G. Yu L. J. Li D. M.. (2019). BYPASS1-LIKE, a DUF793 family protein, participates in freezing tolerance *via* the CBF pathway in *Arabidopsis*. Front. Plant Sci. 10:807. doi: 10.3389/fpls.2019.00807, PMID: 31297122 PMC6607965

[ref10] Chen L. Q. Cheung L. S. Feng L. Tanner W. Frommer W. B. (2015b). Transport of sugars. Annu. Rev. Biochem. 84, 865–894. doi: 10.1146/annurev-biochem-060614-033904 25747398

[ref11] Chen L. Q. Hou B. H. Lalonde S. Takanaga H. Hartung M. L. Qu X. Q.. (2010). Sugar transporters for intercellular exchange and nutrition of pathogens. Nature 468, 527–532. doi: 10.1038/nature09606, PMID: 21107422 PMC3000469

[ref12] Chen H. Y. Huh J. H. Yu Y. C. Ho L. H. Chen L. Q. Tholl D.. (2015a). The Arabidopsis vacuolar sugar transporter SWEET2 limits carbon sequestration from roots and restricts *Pythium* infection. Plant J. 83, 1046–1058. doi: 10.1111/tpj.12948, PMID: 26234706

[ref13] Chen L. Q. Lin I. W. Qu X. Q. Sosso D. McFarlane H. E. Londono A.. (2015c). A cascade of sequentially expressed sucrose transporters in the seed coat and endosperm provides nutrition for the Arabidopsis embryo. Plant Cell 27, 607–619. doi: 10.1105/tpc.114.134585, PMID: 25794936 PMC4558658

[ref14] Chen L. Q. Qu X. Q. Hou B. H. Sosso D. Osorio S. Fernie A. R.. (2012). Sucrose efflux mediated by SWEET proteins as a key step for phloem transport. Science 335, 207–211. doi: 10.1126/science.1213351, PMID: 22157085

[ref15] Cheng J. Wang Z. Yao F. Gao L. Ma S. Sui X.. (2015). Down-regulating CsHT1, a cucumber pollen-specific hexose transporter, inhibits pollen germination, tube growth, and seed development. Plant Physiol. 168, 635–647. doi: 10.1104/pp.15.00290, PMID: 25888616 PMC4453785

[ref16] Chong J. Piron M. C. Meyer S. Merdinoglu D. Bertsch C. Mestre P. (2014). The SWEET family of sugar transporters in grapevine: VvSWEET4 is involved in the interaction with *Botrytis cinerea*. J. Exp. Bot. 65, 6589–6601. doi: 10.1093/jxb/eru375, PMID: 25246444

[ref17] Eom J. S. Chen L. Q. Sosso D. Julius B. T. Lin I. W. Qu X. Q.. (2015). SWEETs, transporters for intracellular and intercellular sugar translocation. Curr. Opin. Plant Biol. 25, 53–62. doi: 10.1016/j.pbi.2015.04.005, PMID: 25988582

[ref18] Feng C. Y. Han J. X. Han X. X. Jiang J. (2015). Genome-wide identification, phylogeny, and expression analysis of the *SWEET* gene family in tomato. Gene 573, 261–272. doi: 10.1016/j.gene.2015.07.055, PMID: 26190159

[ref19] Ge H. Zhang J. Zhang Y. J. Li X. Yin X. R. Grierson D.. (2017). EjNAC3 transcriptionally regulates chilling-induced lignification of loquat fruit *via* physical interaction with an atypical CAD-like gene. J. Exp. Bot. 68, 5129–5136. doi: 10.1093/jxb/erx330, PMID: 28992345 PMC5853329

[ref20] Gilmour S. J. Fowler S. G. Thomashow M. F. (2004). Arabidopsis transcriptional activators CBF1, CBF2, and CBF3 have matching functional activities. Plant Mol. Biol. 54, 767–781. doi: 10.1023/B:PLAN.0000040902.06881.d4, PMID: 15356394

[ref21] Gilmour S. J. Sebolt A. M. Salazar M. P. Everard J. D. Thomashow M. F. (2000). Overexpression of the Arabidopsis *CBF3* transcriptional ativator mimics multiple biochemical changes associated with cold acclimation. Plant Physiol. 124, 1854–1865. doi: 10.1104/pp.124.4.1854, PMID: 11115899 PMC59880

[ref22] Gratkowska-Zmuda D. M. Kubala S. Sarnowska E. Cwiek P. Oksinska P. Steciuk J.. (2020). The SWI/SNF ATP-dependent chromatin remodeling complex in Arabidopsis responds to environmental changes in temperature-dependent manner. Int. J. Mol. Sci. 21:762. doi: 10.3390/ijms21030762, PMID: 31979421 PMC7037086

[ref23] Guo C. Li H. Xia X. Liu X. Yang L. (2018). Functional and evolution characterization of SWEET sugar transporters in *Ananas comosus*. Biochem. Biophys. Res. Commun. 496, 407–414. doi: 10.1016/j.bbrc.2018.01.024, PMID: 29307830

[ref24] Guo W. J. Nagy R. Chen H. Y. Pfrunder S. Yu Y. C. Santelia D.. (2014). SWEET17, a facilitative transporter, mediates fructose transport across the tonoplast of Arabidopsis roots and leaves. Plant Physiol. 164, 777–789. doi: 10.1104/pp.113.232751, PMID: 24381066 PMC3912105

[ref25] Hsieh T. H. Lee J. T. Yang P. T. Chiu L. H. Charng Y. Y. Wang Y. C.. (2002). Heterology expression of the Arabidopsis *C-repeat/dehydration response element binding factor 1* gene confers elevated tolerance to chilling and oxidative stresses in transgenic tomato. Plant Physiol. 129, 1086–1094. doi: 10.1104/pp.003442, PMID: 12114563 PMC166503

[ref26] Huang H. Xie S. Xiao Q. Wei B. Zheng L. Wang Y.. (2016). Sucrose and ABA regulate starch biosynthesis in maize through a novel transcription factor, ZmEREB156. Sci. Rep. 6:27590. doi: 10.1038/srep27590, PMID: 27282997 PMC4901336

[ref27] Jeena G. S. Kumar S. Shukla R. K. (2019). Structure, evolution and diverse physiological roles of SWEET sugar transporters in plants. Plant Mol. Biol. 100, 351–365. doi: 10.1007/s11103-019-00872-4, PMID: 31030374

[ref28] Julius B. T. Leach K. A. Tran T. M. Mertz R. A. Braun D. M. (2017). Sugar transporters in plants: new insights and discoveries. Plant Cell Physiol. 58, 1442–1460. doi: 10.1093/pcp/pcx090, PMID: 28922744

[ref29] Jung H. Chung P. J. Park S. H. Redillas M. Kim Y. S. Suh J. W.. (2017). Overexpression of *OsERF48* causes regulation of *OsCML16*, a calmodulin-like protein gene that enhances root growth and drought tolerance. Plant Biotechnol. J. 15, 1295–1308. doi: 10.1111/pbi.12716, PMID: 28244201 PMC5595718

[ref30] Klemens P. A. Patzke K. Deitmer J. Spinner L. Le Hir R. Bellini C.. (2013). Overexpression of the vacuolar sugar carrier *AtSWEET16* modifies germination, growth, and stress tolerance in Arabidopsis. Plant Physiol. 163, 1338–1352. doi: 10.1104/pp.113.224972, PMID: 24028846 PMC3813654

[ref31] Knight M. R. Knight H. (2012). Low-temperature perception leading to gene expression and cold tolerance in higher plants. New Phytol. 195, 737–751. doi: 10.1111/j.1469-8137.2012.04239.x, PMID: 22816520

[ref32] Lastdrager J. Hanson J. Smeekens S. (2014). Sugar signals and the control of plant growth and development. J. Exp. Bot. 65, 799–807. doi: 10.1093/jxb/ert474 24453229

[ref33] Le Hir R. Spinner L. Klemens P. A. Chakraborti D. de Marco F. Vilaine F.. (2015). Disruption of the sugar transporters *AtSWEET11* and *AtSWEET12* affects vascular development and freezing tolerance in *Arabidopsis*. Mol. Plant 8, 1687–1690. doi: 10.1016/j.molp.2015.08.007, PMID: 26358680

[ref34] Li X. Guo W. Li J. Yue P. Bu H. Jiang J.. (2020b). Histone acetylation at the promoter for the transcription factor PuWRKY31 affects sucrose accumulation in pear fruit. Plant Physiol. 182, 2035–2046. doi: 10.1104/pp.20.00002, PMID: 32047049 PMC7140945

[ref35] Li J. Qin M. Qiao X. Cheng Y. Li X. Zhang H.. (2017a). A new insight into the evolution and functional divergence of SWEET transporters in Chinese white pear (*Pyrus bretschneideri*). Plant Cell Physiol. 58, 839–850. doi: 10.1093/pcp/pcx025, PMID: 28339862

[ref36] Li D. D. Shi W. Deng X. X. (2002). Agrobacterium-mediated transformation of embryogenic calluses of Ponkan mandarin and the regeneration of plants containing the chimeric ribonuclease gene. Plant Cell Rep. 21, 153–156. doi: 10.1007/s00299-002-0492-6

[ref37] Li S. J. Wang W. L. Ma Y. C. Liu S. C. Grierson D. Yin X. R.. (2020a). Citrus CitERF6 contributes to citric acid degradation *via* upregulation of *CitAclalpha1*, encoding ATP-citrate lyase subunit alpha. J. Agric. Food Chem. 68, 10081–10087. doi: 10.1021/acs.jafc.0c03669, PMID: 32820917

[ref38] Li S. J. Xie X. L. Liu S. C. Chen K. S. Yin X. R. (2019). Auto- and mutual-regulation between two *CitERFs* contribute to ethylene-induced citrus fruit degreening. Food Chem. 299:125163. doi: 10.1016/j.foodchem.2019.125163, PMID: 31319344

[ref39] Li S. Xu H. Ju Z. Cao D. Zhu H. Fu D.. (2018). The *RIN-MC* fusion of MADS-box transcription factors has transcriptional activity and modulates expression of many ripening genes. Plant Physiol. 176, 891–909. doi: 10.1104/pp.17.01449, PMID: 29133374 PMC5761797

[ref40] Li S. J. Yin X. R. Wang W. L. Liu X. F. Zhang B. Chen K. S. (2017b). Citrus CitNAC62 cooperates with CitWRKY1 to participate in citric acid degradation *via* up-regulation of *CitAco3*. J. Exp. Bot. 68, 3419–3426. doi: 10.1093/jxb/erx187, PMID: 28633340 PMC5853897

[ref41] Licausi F. Ohme-Takagi M. Perata P. (2013). APETALA2/ethylene responsive factor (AP2/ERF) transcription factors: mediators of stress responses and developmental programs. New Phytol. 199, 639–649. doi: 10.1111/nph.12291, PMID: 24010138

[ref42] Lin I. W. Sosso D. Chen L. Q. Gase K. Kim S. G. Kessler D.. (2014). Nectar secretion requires sucrose phosphate synthases and the sugar transporter SWEET9. Nature 508, 546–549. doi: 10.1038/nature13082, PMID: 24670640

[ref43] Liu F. Xiao Z. Yang L. Chen Q. Shao L. Liu J.. (2017). PhERF6, interacting with EOBI, negatively regulates fragrance biosynthesis in petunia flowers. New Phytol. 215, 1490–1502. doi: 10.1111/nph.14675, PMID: 28675474

[ref44] Liu X. Zhang Y. Yang C. Tian Z. Li J. (2016). AtSWEET4, a hexose facilitator, mediates sugar transport to axial sinks and affects plant development. Sci. Rep. 6:24563. doi: 10.1038/srep24563, PMID: 27102826 PMC4840376

[ref45] Ma L. Zhang D. Miao Q. Yang J. Xuan Y. Hu Y. (2017). Essential role of sugar transporter OsSWEET11 during the early stage of rice grain filling. Plant Cell Physiol. 58, 863–873. doi: 10.1093/pcp/pcx040, PMID: 28371825

[ref46] Patil G. Valliyodan B. Deshmukh R. Prince S. Nicander B. Zhao M.. (2015). Soybean (*Glycine max*) SWEET gene family: insights through comparative genomics, transcriptome profiling and whole genome re-sequence analysis. BMC Genomics 16:520. doi: 10.1186/s12864-015-1730-y, PMID: 26162601 PMC4499210

[ref47] Rogozin I. B. Sverdlov A. V. Babenko V. N. Koonin E. V. (2005). Analysis of evolution of exon-intron structure of eukaryotic genes. Brief. Bioinform. 6, 118–134. doi: 10.1093/bib/6.2.118 15975222

[ref48] Ruan Y. L. (2014). Sucrose metabolism: gateway to diverse carbon use and sugar signaling. Annu. Rev. Plant Biol. 65, 33–67. doi: 10.1146/annurev-arplant-050213-040251, PMID: 24579990

[ref49] Ruan Y. L. Jin Y. Yang Y. J. Li G. J. Boyer J. S. (2010). Sugar input, metabolism, and signaling mediated by invertase: roles in development, yield potential, and response to drought and heat. Mol. Plant 3, 942–955. doi: 10.1093/mp/ssq044, PMID: 20729475

[ref50] Shammai A. Petreikov M. Yeselson Y. Faigenboim A. Moy-Komemi M. Cohen S.. (2018). Natural genetic variation for expression of a SWEET transporter among wild species of *Solanum lycopersicum* (tomato) determines the hexose composition of ripening tomato fruit. Plant J. 96, 343–357. doi: 10.1111/tpj.14035, PMID: 30044900

[ref51] Slewinski T. L. (2011). Diverse functional roles of monosaccharide transporters and their homologs in vascular plants: a physiological perspective. Mol. Plant 4, 641–662. doi: 10.1093/mp/ssr051, PMID: 21746702

[ref52] Streubel J. Pesce C. Hutin M. Koebnik R. Boch J. Szurek B. (2013). Five phylogenetically close rice *SWEET* genes confer TAL effector-mediated susceptibility to *Xanthomonas oryzae* pv. *Oryzae*. New Phytol. 200, 808–819. doi: 10.1111/nph.12411, PMID: 23879865

[ref53] Sun W. Gao Z. Wang J. Huang Y. Chen Y. Li J.. (2019b). Cotton fiber elongation requires the transcription factor GhMYB212 to regulate sucrose transportation into expanding fibers. New Phytol. 222, 864–881. doi: 10.1111/nph.15620, PMID: 30506685

[ref54] Sun L. Sui X. Lucas W. J. Li Y. Feng S. Ma S.. (2019a). Down-regulation of the sucrose transporter *CsSUT1* causes male sterility by altering carbohydrate supply. Plant Physiol. 180, 986–997. doi: 10.1104/pp.19.00317, PMID: 30967482 PMC6548282

[ref55] Vogel M. O. Gomez-Perez D. Probst N. Dietz K. J. (2012). Combinatorial signal integration by APETALA2/ethylene response factor (ERF)-transcription factors and the involvement of AP2-2 in starvation response. Int. J. Mol. Sci. 13, 5933–5951. doi: 10.3390/ijms13055933, PMID: 22754341 PMC3382747

[ref56] Wang S. Yokosho K. Guo R. Whelan J. Ruan Y. L. Ma J. F.. (2019). The soybean sugar transporter GmSWEET15 mediates sucrose export from endosperm to early embryo. Plant Physiol. 180, 2133–2141. doi: 10.1104/pp.19.00641, PMID: 31221732 PMC6670074

[ref57] Wei X. Liu F. Chen C. Ma F. Li M. (2014). The *Malus domestica* sugar transporter gene family: identifications based on genome and expression profiling related to the accumulation of fruit sugars. Front. Plant Sci. 5:569. doi: 10.3389/fpls.2014.00569, PMID: 25414708 PMC4220645

[ref58] Wei L. Mao W. Jia M. Xing S. Ali U. Zhao Y.. (2018). FaMYB44.2, a transcriptional repressor, negatively regulates sucrose accumulation in strawberry receptacles through interplay with FaMYB10. J. Exp. Bot. 69, 4805–4820. doi: 10.1093/jxb/ery249, PMID: 30085079 PMC6137983

[ref59] Wingler A. Juvany M. Cuthbert C. Munne-Bosch S. (2015). Adaptation to altitude affects the senescence response to chilling in the perennial plant *Arabis alpina*. J. Exp. Bot. 66, 355–367. doi: 10.1093/jxb/eru426, PMID: 25371506 PMC4265169

[ref60] Wu Y. Lee S. K. Yoo Y. Wei J. Kwon S. Y. Lee S. W.. (2018). Rice transcription factor OsDOF11 modulates sugar transport by promoting expression of *sucrose transporter* and *SWEET* genes. Mol. Plant 11, 833–845. doi: 10.1016/j.molp.2018.04.002, PMID: 29656028

[ref61] Xie Z. Nolan T. M. Jiang H. Yin Y. (2019). AP2/ERF transcription factor regulatory networks in hormone and abiotic stress responses in *Arabidopsis*. Front. Plant Sci. 10:228. doi: 10.3389/fpls.2019.00228, PMID: 30873200 PMC6403161

[ref62] Xu G. Guo C. Shan H. Kong H. (2012). Divergence of duplicate genes in exon-intron structure. Proc. Natl. Acad. Sci. U. S. A. 109, 1187–1192. doi: 10.1073/pnas.1109047109, PMID: 22232673 PMC3268293

[ref63] Yang J. Luo D. Yang B. Frommer W. B. Eom J. S. (2018). SWEET11 and 15 as key players in seed filling in rice. New Phytol. 218, 604–615. doi: 10.1111/nph.15004, PMID: 29393510

[ref64] Yuan M. Wang S. (2013). Rice MtN3/saliva/SWEET family genes and their homologs in cellular organisms. Mol. Plant 6, 665–674. doi: 10.1093/mp/sst035, PMID: 23430047

[ref65] Yuan M. Zhao J. Huang R. Li X. Xiao J. Wang S. (2014). Rice *MtN3/saliva/SWEET* gene family: evolution, expression profiling, and sugar transport. J. Integr. Plant Biol. 56, 559–570. doi: 10.1111/jipb.12173, PMID: 24456138

[ref66] Zhang J. Yin X. R. Li H. Xu M. Zhang M. X. Li S. J.. (2020). ETHYLENE RESPONSE FACTOR39-MYB8 complex regulates low-temperature-induced lignification of loquat fruit. J. Exp. Bot. 71, 3172–3184. doi: 10.1093/jxb/eraa085, PMID: 32072171 PMC7475177

[ref67] Zhang Y. Yin X. Xiao Y. Zhang Z. Li S. Liu X.. (2018). An ethylene response factor-MYb transcription complex regulates furaneol biosynthesis by activating *QUINONE OXIDOREDUCTASE* expression in strawberry. Plant Physiol. 178, 189–201. doi: 10.1104/pp.18.00598, PMID: 29987002 PMC6130037

