# Supplementary material for: Transcription Factor CitERF16 Is Involved in Citrus Fruit Sucrose Accumulation by Activating CitSWEET11d
Source: Front Plant Sci. 2021 Dec 23;12:809619. doi: 10.3389/fpls.2021.809619 (PMC8733390; doi:10.3389/fpls.2021.809619)
Supplement: Supplementary file 1 [file Data_Sheet_1.docx]

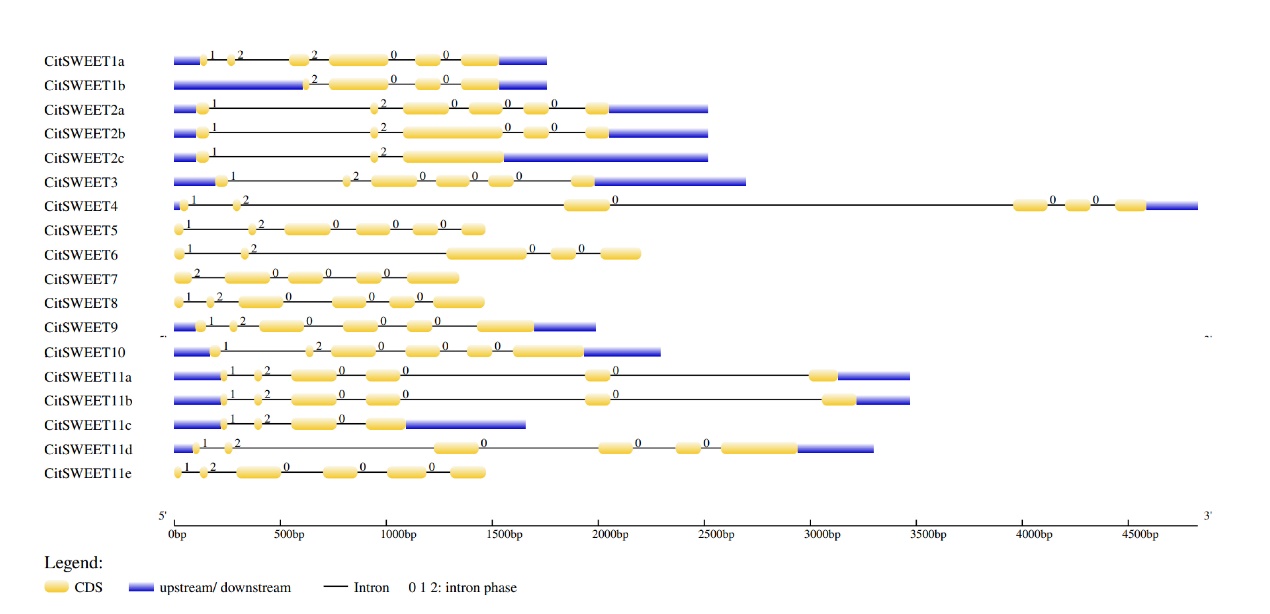


**Supplemental Figure S1.** **Exon-intron structure analysis of 18 *SWEET* genes identified in citrus.** The coding sequence (CDS) is indicated in yellow boxes; the blue boxes represent upstream/downstream regions; the lines between CDS denote introns. Phase 0 indicates introns are positioned between two codons; phase 1 indicates introns insert between the first and the second base of a codon; phase 2 indicates introns insert between the second and the third base of a codon.


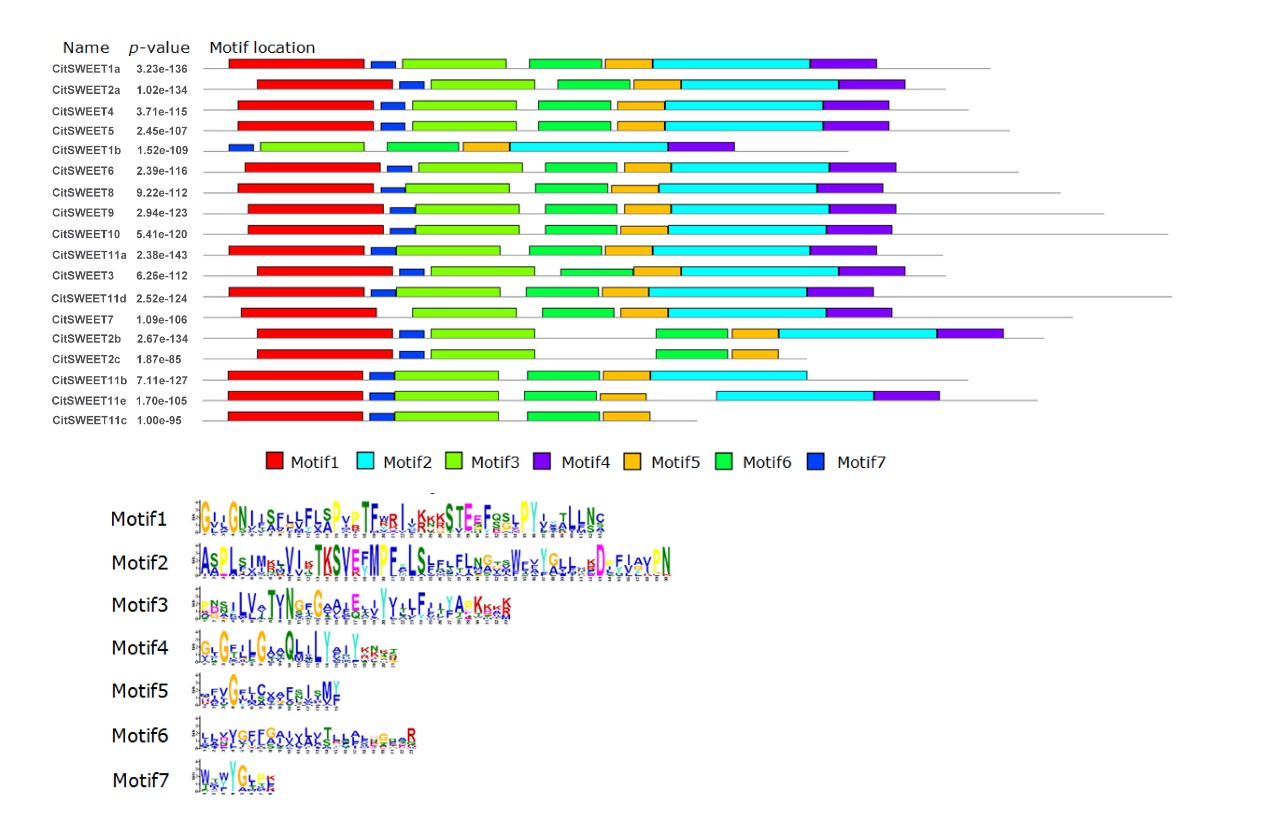


**Supplemental Figure S2. The distribution and sequences of conserved motifs in the citrus *SWEET* family members.** Different colors denote different motifs. The core sequences of each motif are listed below. The alphabet indicates the conserved amino acids residues, and bigger alphabets suggest higher conservative.


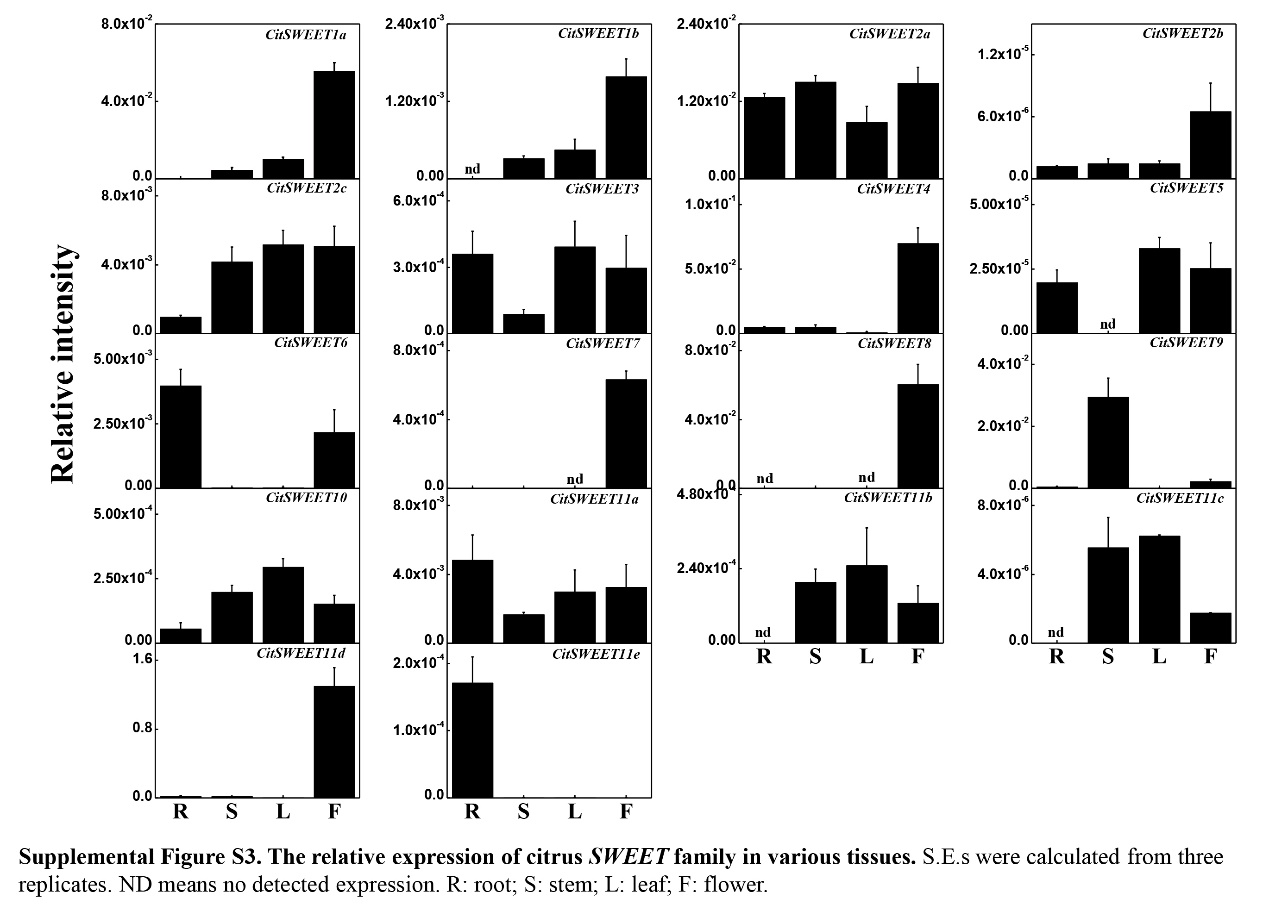


**Supplemental Figure S3.** **The relative expression of citrus *SWEET* family in various tissues.** S.E.s were calculated from three replicates. Nd means no detected expression. R: root; S: stem; L: leaf; F: flower.


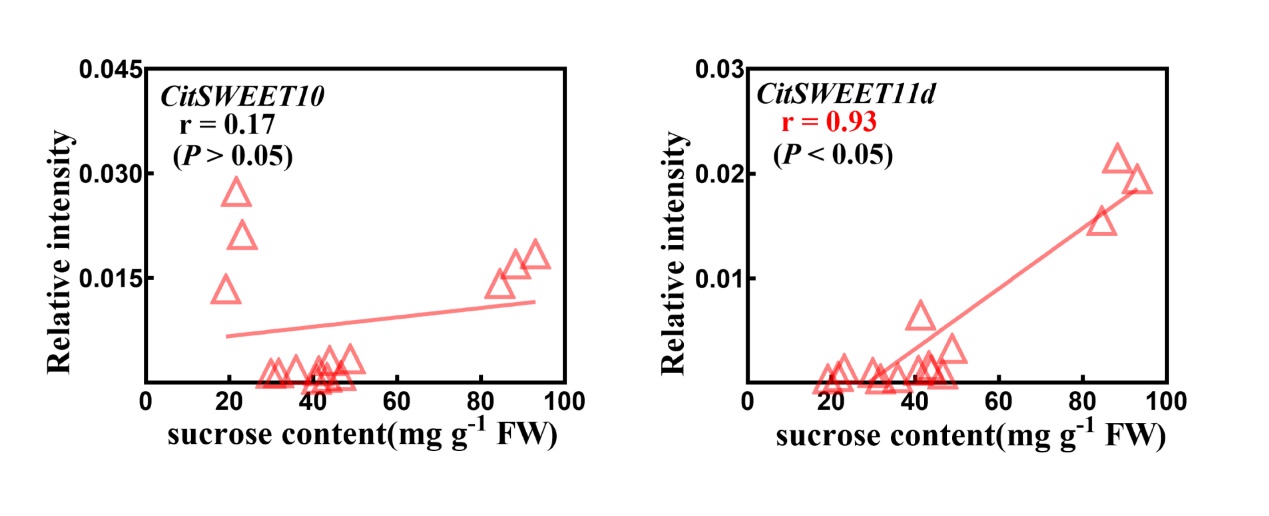


**Supplemental Figure S4.** **Scatterplots between sucrose content and gene expression.** Linear regression analysis between *CitSWEET10* expression as well as *CitSWEET11d* expression and sucrose content. The significant correlation coefficient is denoted in red. Significant diﬀerences were determined by SPSS Statistics 20.0. FW, fresh weight.


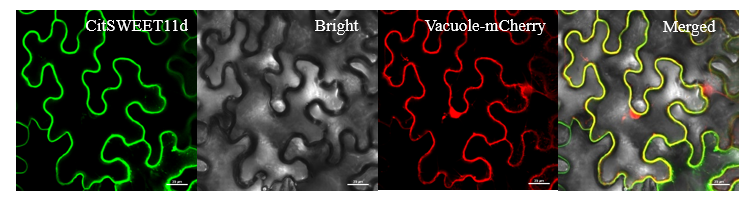


**Supplemental Figure S5.** **The subcellular localization of CitSWEET11d.**


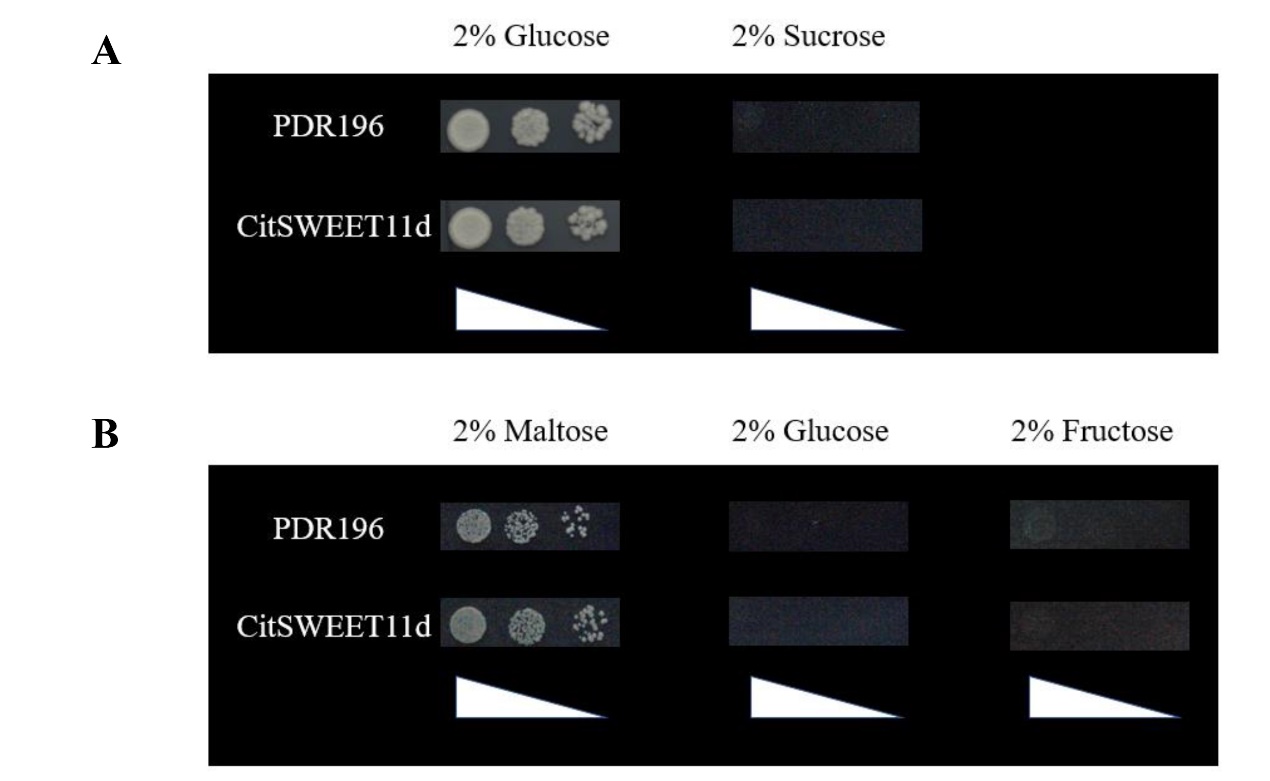


**Supplemental Figure S6.** **The heterologous expression of *CitSWEET11d* in yeast strains SUSY7/*ura* (A) and EBY.VW4000 (B).** Yeast cells containing empty PDR196 vector served as the control. The hypotenuse of the right triangle represents the increase of dilution.


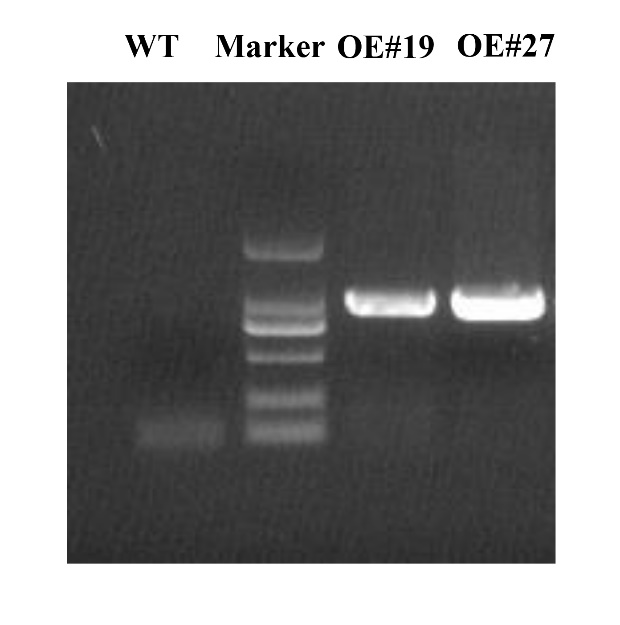


**Supplemental Figure S7. The PCR analysis of wild-type and overexpressed *CitSWEET11d* tomato plants.** The genome DNA was used as the template. The maximum length of marker is 2000bp and the coding sequence length of *CitSWEET11d* is 927bp.


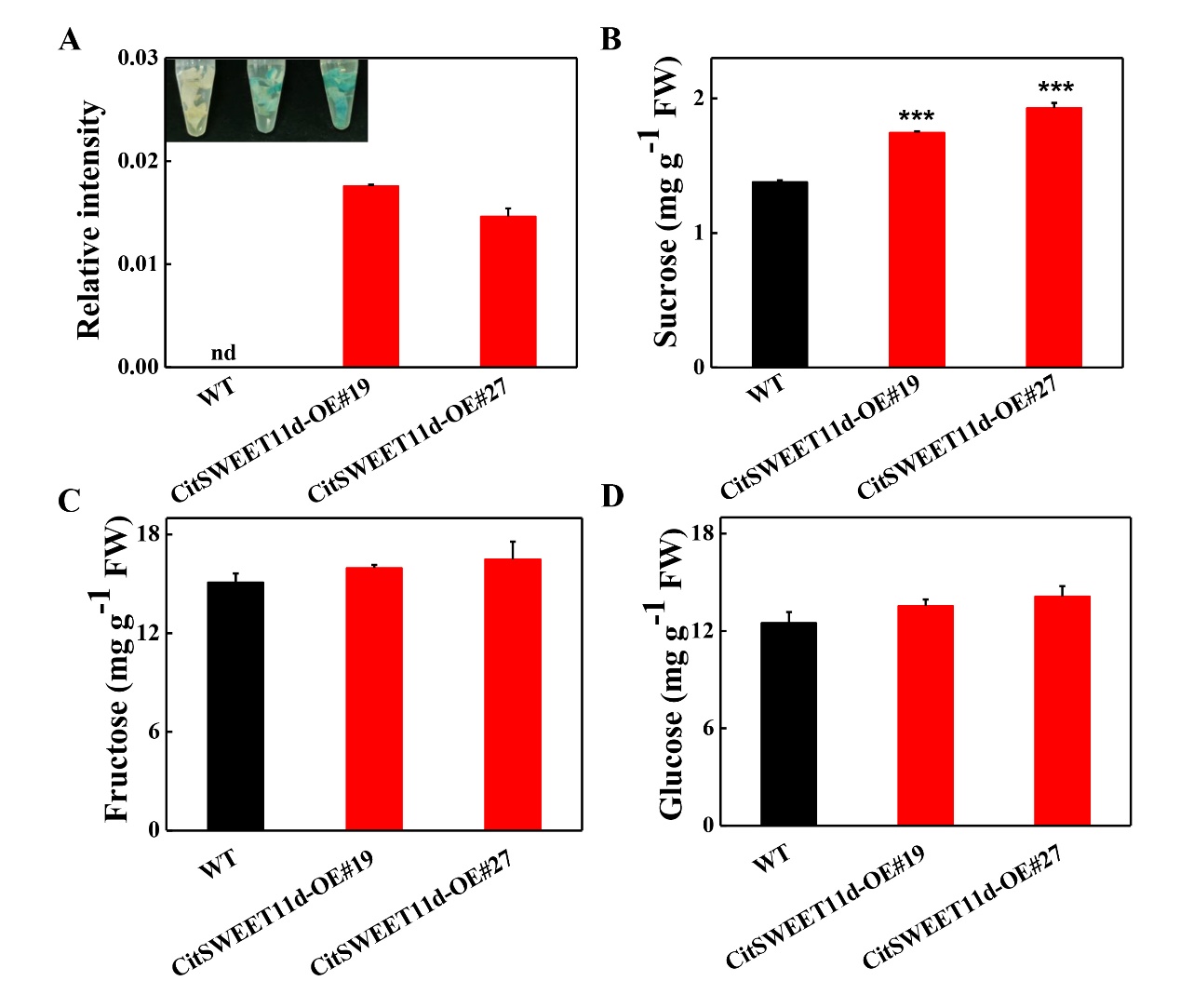


**Supplemental Figure S8.** **Overexpression of *CitSWEET11d* in tomato.** **(A)** GUS staining and qRT-PCR analysis in WT and two independent transgenic tomato lines. **(B)** Sucrose contents of WT and *CitSWEET11d* transgenic fruits at break 7d (seven days after break) stage. **(C)** Fructose contents of WT and *CitSWEET11d* transgenic fruits at break 7d stage. **(D)** Glucose contents of WT and *CitSWEET11d* transgenic fruits at break 7d stage. FW, fresh weight. SEs were calculated from three replicates. Statistical significance was determined by Student’s two-tailed t test (***, *P* < 0.001).


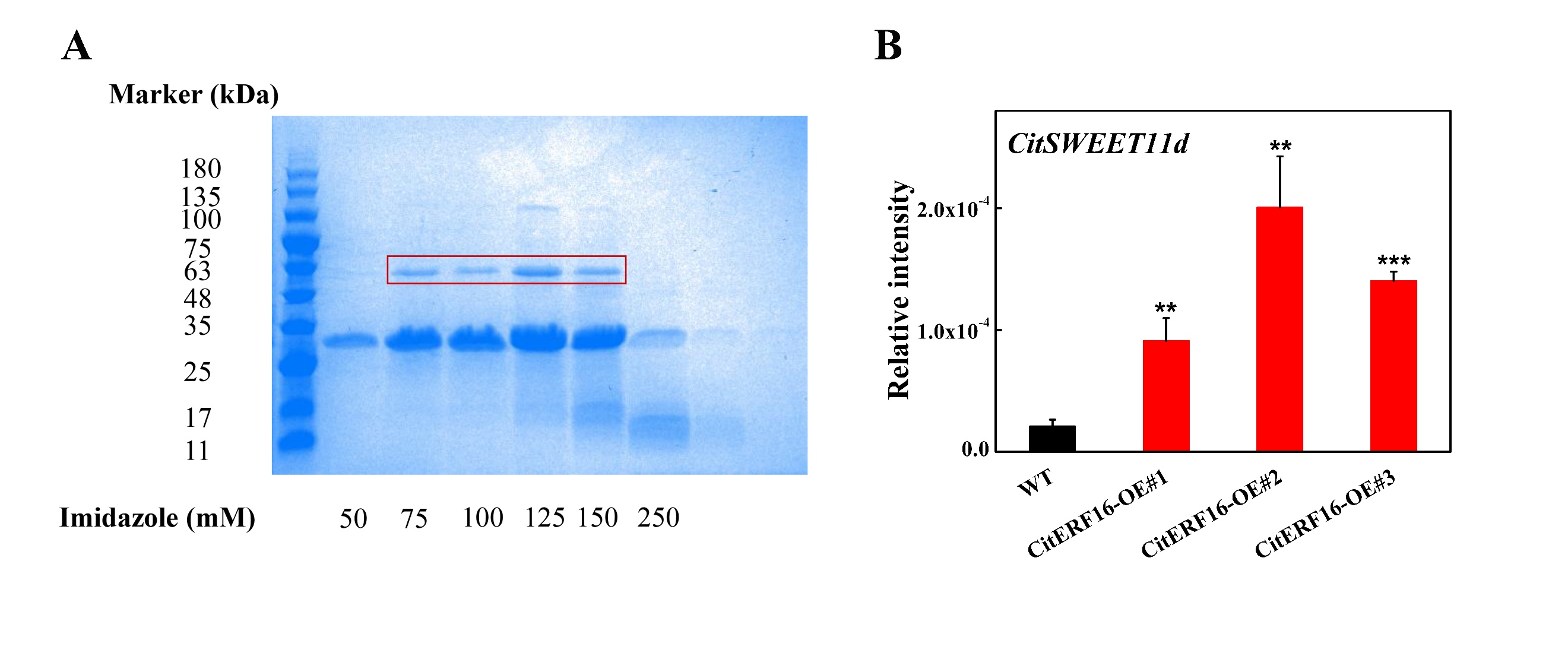


**Supplemental Figure S9.** **(A) CitERF16 protein purification.** Different lanes represent different concentrations of imidazole elution buffer. The proteins in 125 mM imidazole elution buffer were used in EMSA assays. The red box indicates target protein. **(B) The expression level of *CitSWEET11d* in wild-type (WT) and callus overexpressing *CitERF16*.** Error bars indicate the SEs from three replicates. Statistical significance was determined by Student’s two-tailed t test (**, *P* < 0.01; ***, *P* < 0.001).
