# Supplementary material for: Transcription Factor CitERF16 Is Involved in Citrus Fruit Sucrose Accumulation by Activating CitSWEET11d
Source: Front Plant Sci. 2021 Dec 23;12:809619. doi: 10.3389/fpls.2021.809619 (PMC8733390; doi:10.3389/fpls.2021.809619)
Supplement: Supplementary file 2 [file Data_Sheet_2.docx]

**Supplemental Table S1.** Numbers of plant SWEETs in different clades.

| Clade | Ⅰ | Ⅱ | Ⅲ | Ⅳ | total |
| --- | --- | --- | --- | --- | --- |
| *Arabidopsis thaliana* | 3 | 5 | 7 | 2 | 17 |
| *Oryza sativa* | 6 | 9 | 5 | 1 | 21 |
| *Musa acuminata* | 4 | 8 | 10 | 3 | 25 |
| *Triticum aestivum* | 10 | 25 | 19 | 5 | 59 |
| *Vitis vinifera* | 4 | 4 | 5 | 4 | 17 |
| *Citrus clementina* | 6 | 3 | 4 | 5 | 18 |
| *Cucumis sativus* | 3 | 5 | 6 | 3 | 17 |
| *Malus domestica* | 13 | 4 | 11 | 1 | 29 |

**Supplemental Table S2.** The gene IDs of *SWEET* family in citrus

| Name | Gene ID |
| --- | --- |
| *CitSWEET1a* | Ciclev10002276m |
| *CitSWEET1b* | Ciclev10002517m |
| *CitSWEET2a* | Ciclev10022015m |
| *CitSWEET2b* | Ciclev10021714m |
| *CitSWEET2c* | Ciclev10022403m |
| *CitSWEET3* | Ciclev10016509m |
| *CitSWEET4* | Ciclev10016451m |
| *CitSWEET5* | Ciclev10024120m |
| *CitSWEET6* | Ciclev10024343m |
| *CitSWEET7* | Ciclev10023774m |
| *CitSWEET8* | Ciclev10033645m |
| *CitSWEET9* | Ciclev10032313m |
| *CitSWEET10* | Ciclev10032218m |
| *CitSWEET11a* | Ciclev10005775m |
| *CitSWEET11b* | Ciclev10005737m |
| *CitSWEET11c* | Ciclev10006116m |
| *CitSWEET11d* | Ciclev10001944m |
| *CitSWEET11e* | Ciclev10003933m |

**Supplemental Table S3.** Primers used for reverse transcription quantitative PCR.

| **Gene** | **Forward (5’ to 3’)** | **Reverse (5’ to 3’)** |
| --- | --- | --- |
| *CitSWEET1a*  *CitSWEET1b*  *CitSWEET2a*  *CitSWEET2b*  *CitSWEET2c*  *CitSWEET3*  *CitSWEET4 CitSWEET5*  *CitSWEET6*  *CitSWEET7*  *CitSWEET8*  *CitSWEET9*  *CitSWEET10*  *CitSWEET11a*  *CitSWEET11b*  *CitSWEET11c*  *CitSWEET11d*  *CitSWEET11e*  *CitERF16*  *CitActin*  *SlActin* | TCTTGGCTCCAACAATCACA  GGGTAACCGAGAAAGCAGTT  TATGGCACGCCCCTTGTATC  TGCTCGGGTTCTGCAATATG  CTCACGGCAGATGTTTGTTGG  GTGGCAGAGAAGTTAGCGAT  ACTGCCCGGAACATTGTAGG  TGTTGGGATAATCGCCGTTGTCT  GTATGGGTGTTGTACGGGCT  TAGCGAGATATTGCCGGTGG  ACGGAAGGGTTTCAATCGGT  GTCAGTGTTTTTGCCGCACC  AGGAGACCGATCAAAGAAATCCG  ACAAAGAGTGTGGAGTTCAT  TCCTTGGTAAATGCAAAGCCATC  ACTGTCCAAACTGCCCCATT  GGTTAAGACGGCGAAACTTG  AGTGGCTTTAATCATGGAAATGAA  AACGATGCAAGAAAACACATTG  CATCCCTCAGCACCTTCC  ACCTTTGCTGAATACCCTCCATTG | GAGACAATTAAGCAAGGTCATCAC  GACCATACCAGGCAGAGAGG  AGCATCCGCACCTTTTTATCT  TGACCAGATTAATTATGAAC  TAGAGAAAACCACATGCAATACGTT  CCTCAAGTAGTGAAGCGCCTA  CGGGTCTGGCTTGAACTCTT  GCACCATTCGCCGCATTACC  TTAGCACAAGAAGGGCGACA  TCAGGCGGTGTGGTTGATTT  GCATGACGCAGCAGAAAGTG  CGGCATTCAGTGTGAGGAAC  GAAATGACCAAGAAGAGGCGC  GAGCACCAACTGAGCTGTTC  TCACCTCCCTATCGTTTGCG  GTGAGAGGATCTGGGAAGTG  CATGCCAATGGTTAATGCAG  ACTGTCCAAACTGCCCCATT  TCGTCAAATTGAATGACTCCAC  CCAACCTTAGCACTTCTCC  CACAGTTCACTTCCCCTTCTTCTG |

**Supplemental Table S4.** Primers used for vector construction.

| **Primer name Sequence (5'-3')** |
| --- |
| LUC**:**  CitSWEET11d pro-LUC-FP-Sal Ⅰ GGGCCCCCCCTCGAG**GTCGAC**ATAAGAAGAGAAAATTAAGGT  CitSWEET11d pro-LUC-RP-Nco Ⅰ TGTTTTTGGCGTCTT**CCATGG**GGAAGAACTAGAAGAGAATAAAG  pAbAi:  CitSWEET11d pro-pAbAi-FP-Sac Ⅰ GAAAAGCTTGAATTC**GAGCTC**ATAAGAAGAGAAAATTAAGGT  CitSWEET11d pro-pAbAi-RP-Xho Ⅰ ATACAGAGCACATGC**CTCGAG**GGAAGAACTAGAAGAGAATAAAG  GFP:  CitSWEET11d-GFP-FP-BamH Ⅰ CGC**GGATCC**ATGGCTAGCTTAAGCTTCTTCGTC  CitSWEET11d-GFP-RP-Sal Ⅰ ACT**GTCGAC**TGCATCGACGTCGGAATCGTC  1301:  CitSWEET11d-1301-FP-BamH Ⅰ TATC**GGATCC**ATGGCTAGCTTAAGCTTCTTCGTC  CitSWEET11d-1301-RP-Sal Ⅰ GCCA**GTCGAC**TGCATCGACGTCGGAATCGTC  PBTEX:  CitSWEET11d-PBTEX-FP-BamH Ⅰ GGACAGGGTACCCGG**GGATCC**ATGGCTAGCTTAAGCTTCTTCGTC  CitSWEET11d-PBTEX-RP-Sal Ⅰ AGGGCATGCCTGCAG**GTCGAC**TGCATCGACGTCGGAATCGTC  CitERF16-PBTEX-FP-BamH Ⅰ GGACAGGGTACCCGG**GGATCC**ATGGACATATTATTCGGCCAAG  CitERF16-PBTEX-RP-Sal Ⅰ AGGGCATGCCTGCAG**GTCGAC**TCAAATTGAATGACTCCACAAC  pET32a:  CitERF16-pET32a-FP-BamH Ⅰ GCCATGGCTGATATC**GGATCC**ATGGACATATTATTCGGCCAAG  CitERF16-pET32a-RP-Sal Ⅰ TGCGGCCGCAAGCTT**GTCGAC**AATTGAATGACTCCACAACGAC  pGADT7:  CitERF16-AD-FP-EcoR Ⅰ GCCATGGAGGCCAGT**GAATTC**ATGGACATATTATTCGGCCAAG  CitERF16-AD-RP-BamH Ⅰ CAGCTCGAGCTCGAT**GGATCC**TCAAATTGAATGACTCCACAAC  SK:  CitERF16-SK-FP-BamH Ⅰ CGCTCTAGAACTAGT**GGATCC**ATGGACATATTATTCGGCCAAG  CitERF16-SK-RP-Sal Ⅰ GGGCCCCCCCTCGAG**GTCGAC**TCAAATTGAATGACTCCACAAC  PDR196:  CitSWEET11d-PDR196-FP-EcoR Ⅰ TCCCCCGGGCTGCAG**GAATTC**ATGGCTAGCTTAAGCTTCTT  CitSWEET11d-PDR196-RP-Sal Ⅰ GGGCCCCCCCTCGAG**GTCGAC**TCATGCATCGACGTCGGAAT  EMSA:  Probe-FP-biotin: AAACGATGTCGGTACTCGGTCCGCATGCAATCATACAAAACAACA AGAAT  Probe-RP-biotin: ATTCTTGTTGTTTTGTATGATTGCATGCGGACCGAGTACCGACATCGTTT  Probe-FP: AAACGATGTCGGTACTCGGTCCGCATGCAATCATACAAAACAACA AGAAT  Probe-RP: ATTCTTGTTGTTTTGTATGATTGCATGCGGACCGAGTACCGACATCGTTT |

Note: Added restriction enzyme sites are underlined.
